# Fine-Tuned Enzymatic Hydrolysis of Organosolv Pretreated Forest Materials for the Efficient Production of Cellobiose

**DOI:** 10.3389/fchem.2018.00128

**Published:** 2018-04-19

**Authors:** Anthi Karnaouri, Evangelos Topakas, Leonidas Matsakas, Ulrika Rova, Paul Christakopoulos

**Affiliations:** ^1^Biochemical Process Engineering, Chemical Engineering, Department of Civil, Environmental and Natural Resources Engineering, Luleå University of Technology, Luleå, Sweden; ^2^Biotechnology Laboratory, Department of Synthesis and Development of Industrial Processes, School of Chemical Engineering, National Technical University of Athens, Athens, Greece

**Keywords:** cellobiohydrolases, hydrolysis, enzymatic cocktail, cellobiose, experimental design, thermostable enzymes, prebiotics

## Abstract

Non-digestible oligosaccharides (NDOs) are likely prebiotic candidates that have been related to the prevention of intestinal infections and other disorders for both humans and animals. Lignocellulosic biomass is the largest carbon source in the biosphere, therefore cello-oligosacharides (COS), especially cellobiose, are potentially the most widely available choice of NDOs. Production of COS and cellobiose with enzymes offers numerous benefits over acid-catalyzed processes, as it is milder, environmentally friendly and produces fewer by-products. Cellobiohydrolases (CBHs) and a class of endoglucanases (EGs), namely processive EGs, are key enzymes for the production of COS, as they have higher preference toward glycosidic bonds near the end of cellulose chains and are able to release soluble products. In this work, we describe the heterologous expression and characterization of two CBHs from the filamentous fungus *Thermothelomyces thermophila*, as well as their synergism with proccessive EGs for cellobiose release from organosolv pretreated spruce and birch. The properties, inhibition kinetics and substrate specific activities for each enzyme are described in detail. The results show that a combination of EGs belonging to Glycosyl hydrolase families 5, 6, and 9, with a CBHI and CBHII in appropriate proportions, can enhance the production of COS from forest materials, underpinning the potential of these biocatalysts in the production of NDOs.

## Introduction

In food and nutraceutical industry, the development of compounds that have the potential to reduce disease risk and thereby enhance human health has attracted much interest. Currently, one of the main targets of research is the gastrointestinal tract and its resident microbiome (Cummings and Macfarlane, 1991). Diet-related modulations of this microflora offers promise for reducing pathogens/mediated gut disorders linked to a variety of chronic diseases, including obesity, type 2 diabetes, and cardiovascular diseases. This has resulted in the development of a prebiotic concept. Prebiotics are dietary ingredients that are not digested and selectively promote the growth and the activity of the bacteria in the colon (Gibson and Roberfroid, 1995). The level of active prebiotics that can be obtained through the diet is too low to have a significant impact; therefore, a sustainable production of prebiotics with a defined action is to be preferred.

Non-digestible oligosaccharides (NDOs) are oligosaccharides with a degree of polymerization (DP) of 2–9 that are resistant to digestion by human gastric and pancreatic enzymes and seem to be preferred by the gut microbiota. NDOs, especially cellobiose, have attracted much attention since they have low calorific value (Livesey, 1990; Roberfroid et al., 1993), potential prebiotic effect and can add functionality to the food products (e.g., as rheology enhancers, bulking agents, stabilizers; Figueroa-González et al., 2011). The prebiotic potential of cellobiose has been investigated by *in vitro* fermentations in the presence of a human fecal inoculum, where it was shown to increase bifidobacteria, lactobacilli, and short chain fatty acids (SCFA); markers of a potentially beneficial effect in modulating the gut microbiome (van Zanten et al., 2012). Aside from a likely prebiotic candidate, cellobiose has been also used as a sweetener (Kulka and Ungureanu, 2017), a cosmetic additive in its acylated form (Franklin et al., 2002) or a building block in polymers (Berson et al., 2008). Cellobiose and other cello-oligosaccharides (COS) can be obtained by acid-based and enzyme-based hydrolysis of the insoluble cellulose. Enzymatic hydrolysis is considered more attractive due to the relatively mild reaction condition (less by-products) and the easier control of the polysaccharide cleavage breaking (less monomers). A consortium of enzymes is required for the degradation of cellulose, including *endoglucanases* (EG), *cellobiohydrolases* (CBH), and β*-glucosidases* (BGL). In addition, lytic polysaccharide monooxygenases (LPMOs) act in strong synergism with endoglucanases both for the release of neutral and oxidized sugars (Karnaouri et al., 2017). Based on the hydrolysis patterns for each catalytic reaction by cellulases, cellobiose is accumulated when BGL is not present.

*CBHs* are enzymes of pivotal importance for the cellobiose production, as they catalyze the hydrolysis of the β-1,4 bonds at the ends of the cellulose chains releasing mainly cellobiose. They are characterized as CBH I (glycoside hydrolase family 7; GH7) and CBH II (glycoside hydrolase family 6; GH6) and act on cellulose molecules from reducing and non-reducing ends, respectively. The enzymes belonging to each family possess a high identity degree of their amino acid sequence and common folding domains in their structural conformation. CBHII enzymes act through inversion of the anomeric configuration of the substrate, while CBHI enzymes usually retain the configuration (Schülein, 2000). The typical molecular structure for most fungal CBHs includes a core catalytic domain and a cellulose-binding module (CBM) joined by a flexible peptide linker (van Tilbeurgh et al., 1986; Gilkes et al., 1991) and are often glycosylated, involving both *O*- and *N-*linked carbohydrate structures (Maras et al., 1997; Hu et al., 2001). The ability of catalyzing successive cleavage of sugar bonds without detaching from the substrate, called *processivity*, is a common characteristic to CBHs and is considered to boost the catalytic efficiency of the enzyme when acting on substrates with high crystallinity (Teeri, 1997). Structural studies of CBHs isolated from *Trichoderma reesei* have demonstrated that processivity is associated with the existence of a tunnel-shaped active site, where a single glucan can enter, and the cleavage occurs during its passage (Kurasin and Väljamäe, 2011). An exo–exo synergism between CBHI and CBHII that increases the saccharification yields has been reported (Medve et al., 1994).

*EGs* are also key enzymes for the production of COS in many ways. Firstly, a class of them, namely *processive*, that have higher preference toward glycosidic bonds near the end of cellulose chains, are able to release soluble COS (mainly C2 and C4) before detaching from the substrate (Wilson and Kostylev, 2012). In addition, individual cellulolytic activity of EGs has been related to rapid and efficient liquefaction of cellulose-rich lignocellulosic materials, such as wheat straw, under high dry matter loadings [19% (w/w) DM], which is of outmost importance in order to achieve high COS yields (Karnaouri et al., 2014). When CBHs act in concert with the EGs, hydrolysis yields increase drastically due to endo–exo synergy between two classes of the enzymes (Henrissat et al., 1985).

In the present work, we used CBHI and CBHII from *Thermothelomyces thermophila* cloned in *Pichia pastoris*, and tested them in optimized enzyme mixtures with the aim to maximize cellulose conversion into cellobiose, using organosolv pretreated spruce and birch. First, the optimal mixture that maximizes cellobiose production was identified by using different combinations of commercially available enzymes, two EGs and two CBHs. The optimal cocktails were identified via statistically-designed experiments based on cellobiose release. Subsequently, *Tt*CBH6 and *Tt*CBH7 were used to replace the CBHs in optimized combinations in order to evaluate the performance of these *in-house* produced enzymes and compare with that of commercially available CBHs.

## Materials and methods

### Enzymes and chemicals

For the cloning of the CBHs genes, KOD Hot Start® DNA polymerase was purchached from Novagen (USA) and restriction enzymes were from TAKARA (Japan). Nucleospin Gel Clean-up and GeneJET Plasmid Miniprep kits were obtained from Macherey–Nagel (Germany) and Fermentas (USA), respectively. Barley β-glucan, xylooligosaccharides and mannooligosaccharides were purchased from Megazyme, microcrystalline cellulose Avicel PH-101 was from Merck (Darmstadt, Germany) and D-cellobiose was from Fluka. 4-Nitrophenyl β-D-glucopyranoside, 4-Nitrophenyl β-D-lactopyranoside and 4-methylumbelliferyl b-D-cellobioside were from Sigma–Aldrich. All other chemicals used in this study were of analytical grade. Phosphoric acid swollen cellulose (PASC) was prepared from Avicel, following the protocol initially described by Wood (1988).

For the cellobiose production experiements, endo-1,4-β-D-glucanase (EG7) and cellobiohydrolase I (CBH7) from *Trichoderma longibrachiatum*, endo-1,4-β-D-glucanase (EG5) from *Talaromyces emersonii* and cellobiohydrolase II (CBH6) from microbial source were from Megazyme. Cellulase 6A (EG6) from *Podospora anserina*, Cellulase 9A (EG9) from *Clostridium thermocellum* and Cellulase 12B (EG12) from *Thermotoga maritima* were purchased from NZytech (NZYTech, Lda., Portugal). Organosolv-pretreated birch (200°C for 30 min, 60% EtOH) and spruce (200°C for 30 min, 52% EtOH) were used as substrates. The composition of birch (w/w) was 67.1% cellulose, 21% hemicellulose and 7.1% lignin and that of spruce was 66% cellulose, 6% hemicellulose, and 14.9% lignin.

### Cloning of *cbh6* and *cbh7* genes from *T. thermophila*

The host-vector system of One Shot® Top10 *Escherichia coli* cells (Invitrogen, USA) and Zero Blunt® PCR Cloning Kit (Invitrogen, USA) was used for the cloning of the both CBH genes from *T. thermophila* The wild-type strain of *M. thermophila* ATCC 42464 was used. Protein expression was achieved with *P. pastoris* host strain X33 and pPICZαC (Invitrogen, USA). *P. pastoris* was cultivated in shaking flasks at 30°C following the EasySelect™ *Pichia Expression Kit* (Invitrogen, USA) instructions. Genomic DNA was prepared and isolated as previously described (Topakas et al., 2012). The *E. coli*/*P. pastoris* shuttle vector pPICZαC, containing the tightly regulated *AOX1* promoter and the *Saccharomyces cerevisiae* α-factor secretion signal (Higgins et al., 1998), was used for the expression of *Tt*CBH6a and *Tt*CBH7a. The genes coding for the hypothetical proteins *Tt*CBH6 [MYCTH_66729, GenBank: AEO55787.1] and *Tt*CBH7 [MYCTH_109566, GenBank: AEO55544.1] were amplified with PCR from genomic DNA using primers **EF/ER** (Tables S1A, S2A) designed accordingly to the gene sequences (http://genome.jgi-psf.org/, DOE Joint Genome Institute, Berka et al., 2011).

A high fidelity KOD Hot Start® DNA polymerase was used for the DNA amplification, which was carried out with 30 cycles of denaturation (20 s at 95°C), annealing (10 s at 58°C for *Tt*CBH6 and 60°C for *Tt*CBH7), and extension (32 s at 70°C), followed by 1 min of further extension at 70°C (Tables S1B, S2B). In order to determine the DNA sequence, the PCR product, containing exons 2–4 and introns was cloned into the pCRBlunt® vector following the protocol described by the Zero Blunt® PCR Cloning Kit. Intron removal was achieved using the molecular technique of overlap extension polymerase chain reaction (OEPCR; Topakas et al., 2012). For the *Tt*CBH6, two complementary DNA primers per intron (**Ee2F/Ee2R**, **Ee3F/Ee3R**, **Ee4F/ER**, Table S1A) were used to generate two DNA fragments with overlapping ends following the appropriate PCR amplification process and using the recombinant plasmid pCRBlunt/*cbh6* as template. The primer **Ee2F** included the sequence of exon 1, as well as the *ClaI* restriction enzyme site at 5′-end and was used for the synthesis of the *N*-terminal part of the protein in order to avoid overlapping PCR (Figure S1). The annealing and extension conditions for each DNA fragment are described in Table S1B. The three PCR products were combined together in a subsequent hybridization reaction. The generated “*fusion*” fragment was amplified further by overlapping PCR through the utilization of the two external primers, **EF** end **ER**, performing an extended annealing step on order to improve base-pairing between the complementary ends of each fragment. Intron removal of *Tt*CBH7 was achieved using the plasmid pCRBlunt/cbh7 as template, with two pairs of complementary DNA primers (**EF/Ee1R, Ee2F/ER**, Table S2A). The two PCR products harboring overlapping ends were combined together in a subsequent hybridization reaction with **EF** and **ER** primers (Table S2A), as described above. The produced *cbh7* and *cbh6* DNA fragments were digested with the enzymes *ClaI* and *XbaI*, cloned into the pPICZαC vector and amplified in *E. coli* TOP10F'. The recombinant vectors were confirmed by restriction analysis and DNA sequencing and finally transformed into *P. pastoris* according to the EasySelect^TM^ *Pichia* Expression Kit. The production and purification of recombinant *Tt*CBH6 and *Tt*CBH7 enzymes were performed as previously described (Karnaouri et al., 2017).

### Characterization of *Tt*CBH6 and *Tt*CBH7

The activity of *Tt*CBH6 and *Tt*CBH7 was determined on Avicel 5% (w/v) for 1 h, at 50°C in 0.1 M citrate–phosphate buffer pH 5.0. The concentration of reducing ends was determined using the dinitrosalicylic acid reagent (DNS; Miller, 1959) and glucose for the standard curve. One unit (U) of enzymatic activity was defined as the amount of enzyme that released 1 μmol of sugar (glucose equivalents) per minute. Protein concentration was determined by the bicinchoninic acid (BCA) protein assay microplate procedure (Pierce Chemical Co., Rockford, IL), with bovine serum albumin as standard, according to the manufacturer's instructions (Smith et al., 1985). Substrate specificity of pure *Tt*CBH6 and *Tt*CBH7 enzymes was tested against 4-nitrophenyl β-D-cellobioside 5 mM, 4-nitrophenyl β-D-lactopyranoside 5 mM, β-glucan 0.5% w/v, PASC 0.5% w/v and carboxyl-methyl-cellulose (CMC) 1% w/v. Enzyme activity was determined in 0.1 M citrate–phosphate buffer pH 5.0 at 50°C for 15 min. The amount of reducing sugars released from β-glucan, PASC and CMC was estimated using the DNS method, as described above. The formation of 4-nitrophenol was measured at A_410_, after addition of 1 M Na_2_CO_3_ to the reaction mixtures, using a standard curve under the same conditions.

The determination of optimal temperature of *Tt*CBH6 and *Tt*CBH7 was performed using Avicel 5% (w/v) as a substrate, in 0.1 M citrate-phosphate buffer pH 5.0, at temperatures ranging from 30 to 90°C. Temperature stability was determined by measuring the residual activity under the same assay procedure, after incubation of 0.32 and 0.45 mg of purified *Tt*CBH6 and *Tt*CBH7, respectively at various temperatures for different amount of time. pH optimal was estimated at 50°C, over the pH range 3.0–11.0 using either 0.1 M citrate–phosphate buffer pH 3.0–7.0, 0.1 M Tris-HCl pH 7.0–9.0 or 0.1 M glycine–NaOH buffer pH 9.0–11.0. The stability at different pH was determined after incubating the enzymes in the above buffers at 4°C for 24 h and then measuring the activity remaining using the Avicel assay, as described above.

The values of the maximum velocity (V_*max*_) and the Michaelis constant (K_*m*_) for *Tt*CBH6 and *Tt*CBH7 were determined by incubating the enzymes in 0.1 M sodium acetate buffer pH 5.0 at 45°C for 30 min with 4-methyl-umbelliferyl-β-cellobiose (MUG2) at concentrations ranging from 0.1 to 2 mM (Boschker and Cappenberg, 1994). The release of methyl-umbelliferyl was followed by measuring the absorbance at A_350_. The inhibition of CBHs by specific mono/oligosaccharides was determined by estimating the enzymatic activity on MUG2 in presence of different inhibitor concentrations (5 and 10 mM). Kinetic analysis of inhibition was carried out on xylose (X1), xylobiose (X2), xylotriose (X3), mannose (M1), mannobiose (M2), mannotriose (M3), glucose (G1), cellobiose (G2), and xylosyl-cellobiose (XC). Lineweaver–Burk plots were drawn to determine K_*m*_, V_*max*_, and inhibition constant (K_*i*_) values.

### Hydrolysis of organosolv pretreated materials

The release of cellobiose from organosolv-pretreated materials (birch and spruce) with different enzyme mixtures was studied. The four major commercial cellulases EG5, EG7, CBH7, and CBH6 were used to set up an experimental design (**#1**) with the software Design Expert® 7.0.0 (Stat-Ease Inc.), targeting increased % cellobiose yield. The algorithmically built *D-optimal* design was employed to generate 20 experimental conditions (Table 1) where the enzymes varied between specified levels (Table 2). Among these combinations, 5 replicates were included in the design, in order to increase the power of the model and reduce the prediction error. The replicates represented enzyme concentrations that corresponded to the upper and lower limit values. Based on literature data (Billard et al., 2012; Karnaouri et al., 2016) combined with preliminary experimental results, the upper and lower limits of each enzyme and their relative abundance were carefully chosen. In all the experimental combinations, the proportion of each enzyme varied, with the total amount of the enzyme loading to be kept constant and set to be equal to 1 (or 100%). Evaluation of the results and determination of the suitable model that fits the experimental data was done with the same software. The two models applied were either the *quadratic* or the *special cubic* (Karnaouri et al., 2016). The efficiency of the model was evaluated by calculating the *p-*value and *R*^2^. Optimization of the mixture toward maximal cellobiose yields was also performed by the same software. All reactions were performed at 50°C, with 2.5% initial dry matter content, in 0.1 M phosphate-citrate buffer pH 5.0 with 0.02% NaN_3_, in a final volume of 1 mL. The total enzyme loading was 25 mg/g substrate. Samples were taken at 24 and 48 h, filtered and analyzed for the release of cellobiose and glucose with isocratic ion-exchange chromatography using an Aminex HPX-87P column (Bio-Rad Laboratories, Hercules, CA, USA) and Millipore water as the mobile phase.

**Table 1:** Experimental combinations used for the hydrolysis tests generated with *D-optimal* design (Design Expert® 7.0.0, Stat-Ease Inc.).

**Component**	**#1**				**#2**			
	**A**	**B**	**C**	**D**	**A**	**B**	**C**	**D**
**Run**	**EG5**	**EG7**	**CBH6**	**CBH7**	**EG5**	**EG7**	**EG9**	**EG6**
1	0.25	0.2	0.05	0.5	0.38	0.55	0.04	0.03
2	0.1	0.05	0.05	0.8	0.20	0.70	0.06	0.05
3	0.1	0.1	0.3	0.5	0.35	0.50	0.10	0.06
4	0.221	0.05	0.193	0.537	0.20	0.69	0.10	0.01
5	0.3	0.055	0.145	0.5	0.27	0.70	0.02	0.01
6	0.182	0.122	0.135	0.56	0.32	0.55	0.06	0.07
7	0.1	0.2	0.2	0.5	0.25	0.63	0.06	0.05
8	0.1	0.2	0.05	0.65	0.45	0.50	0.02	0.03
9	0.1	0.05	0.211	0.639	0.33	0.60	0.06	0.01
10	0.156	0.102	0.05	0.693	0.45	0.50	0.02	0.03
11	0.175	0.05	0.275	0.5	0.20	0.68	0.02	0.10
12	0.179	0.193	0.05	0.578	0.20	0.60	0.10	0.10
13	0.27	0.05	0.05	0.63	0.20	0.60	0.10	0.10
14	0.176	0.051	0.133	0.64	0.20	0.68	0.02	0.10
15	0.27	0.05	0.05	0.63	0.30	0.50	0.10	0.10
16	0.25	0.2	0.05	0.5	0.38	0.50	0.02	0.10
17	0.1	0.05	0.132	0.718	0.27	0.57	0.06	0.10
18	0.1	0.2	0.2	0.5	0.27	0.70	0.02	0.01
19	0.3	0.055	0.145	0.5	0.33	0.60	0.02	0.06
20	0.1	0.05	0.05	0.8	0.20	0.69	0.10	0.01

**Table 2:** Upper and lower constrains for all variables used for the experimental design #1 and #2.

	**Variable in model**	**Lower limit**	**Upper limit**
**#1**			
EG5	A	0.1	0.2
EG7	B	0.05	0.2
CBH6	C	0.05	0.3
CBH7	D	0.5	0.8
**#2**			
EG5	A	0.2	0.45
CBH7	B	0.5	0.7
EG9	C	0.02	0.1
EG6	D	0.01	0.1

After identifying the optimal enzyme combination that maximizes cellobiose production, another experimental design (**#2**) was set up using different proportions of EG5, CBH7, EG9, and EG6, while the CBH6 and EG7 were kept constant and equal with those values that gave the maximal cellobiose concentration and % yield in design **#1**. Data analysis, evaluation of the model and optimization of the mixture were performed as described above. The theoretically predicted highest yields in both experimental designs were verified with time-course experiments. *Tt*CBH6 and *Tt*CBH7 were finally used to replace CBH7 and CBH6 in optimized combinations in order to evaluate the performance of these in-house produced enzymes and compare with that of commercially available cellobiohydrolases.

## Results

### Heterologous expression and characterization of *Tt*CBH6 and *Tt*CBH7

From genome analysis, the translation of *cbh6* and *cbh7* open reading frames (ORF) from the *T. thermophila* genome database show significant primary sequence identity with characterized CBHs acting on the non-reducing and the reducing end of the carbohydrate molecules, which have been classified to GH families 6 and 7, respectively, on CAZy database (http://www.cazy.org/; Cantarel et al., 2009; Terrapon et al., 2017). The putative cellobiohydrolase *Tt*CBH6 showed high sequence identity (79%) with the CBHII from *Humicola insolens* [PDB ID: 1BVW] and 64% with the CBHII from *Trichoderma viride* [GenBank: AAQ76094.1]. The putative *Tt*CBH7 showed high sequence identity (67%) with the Cel7d (Cbh58) *Phanerochaete chrysosporium* [PDB: 1GPI] and 61% identity with the CBH I from *Humicola grisea* [GenBank: BAA09785.1]. The hypothetical proteins of 66729 and 109566 were selected as putative CBHs and the corresponding genes, provisionally named *cbh6* and *cbh7*, were cloned and used to transform *P. pastoris* X33, encoding T*t*CBH6 and *Tt*CBH7, respectively (Table S3). The ORF of *cbh6* encodes a protein of 465 amino acids including a secretion signal peptide of 17 amino acids (MAKKLFITAALAAAVLA) based upon the prediction using SignalP v4.0 (http://www.cbs.dtu.dk/services/SignalP/). The predicted mass and isoelectric point (pI) of the mature protein is 49.41 kDa and pI 5.28, respectively, by calculations using the ProtParam tool of ExPASY (http://web.expasy.org/protparam/). The ORF of *cbh7* encodes a protein of 509 amino acids including a secretion signal peptide of 17 amino acids (MYAKFATLAALVAGAAA), while the predicted mass and isoelectric point (pI) of the mature protein is 54 kDa and pI 4.77.

**Figure 1:**
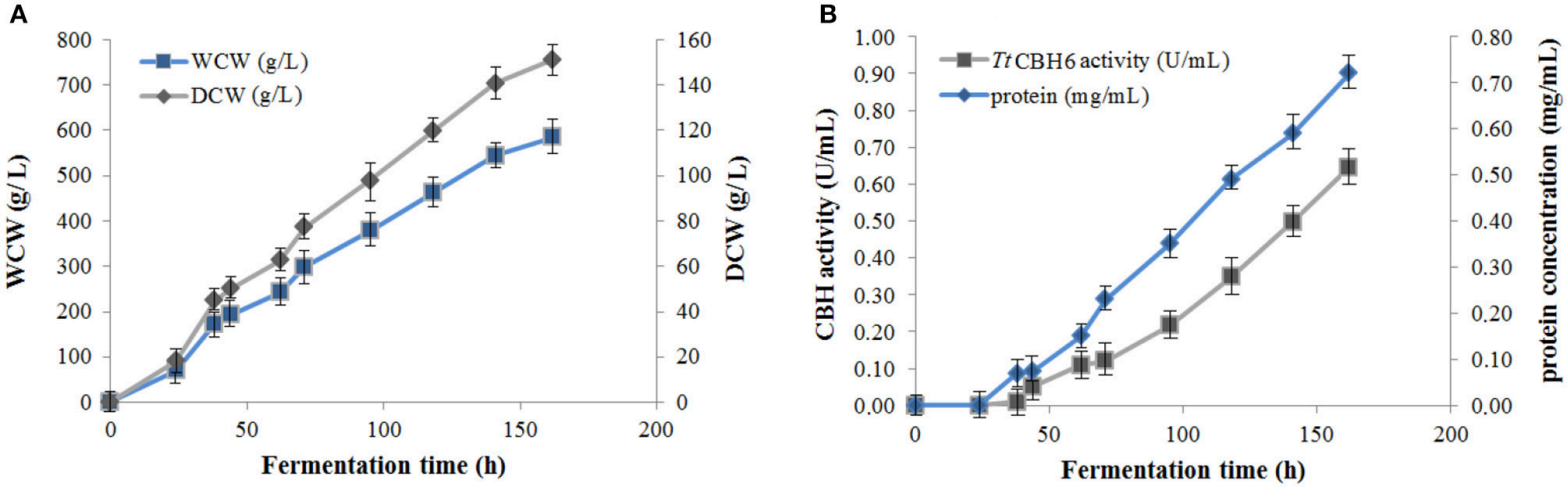
**(A)** Cell mass concentration during *Tt*CBH6 fermentation. Dry cell weight (DCW) reached 45 g/L after glycerol fed-batch phase and 157 g/L at the end of the cultivation, corresponding to wet cell weight (WCW) of 290 and 580 g/L, respectively. **(B)** Protein concentration and CBH activity detected in the culture medium during fermentation. Specific activity was tested against Avicel 5% (w/v), pH 5.0, 50°C in 100 mM phosphate-citrate buffer.

**Figure 2:**
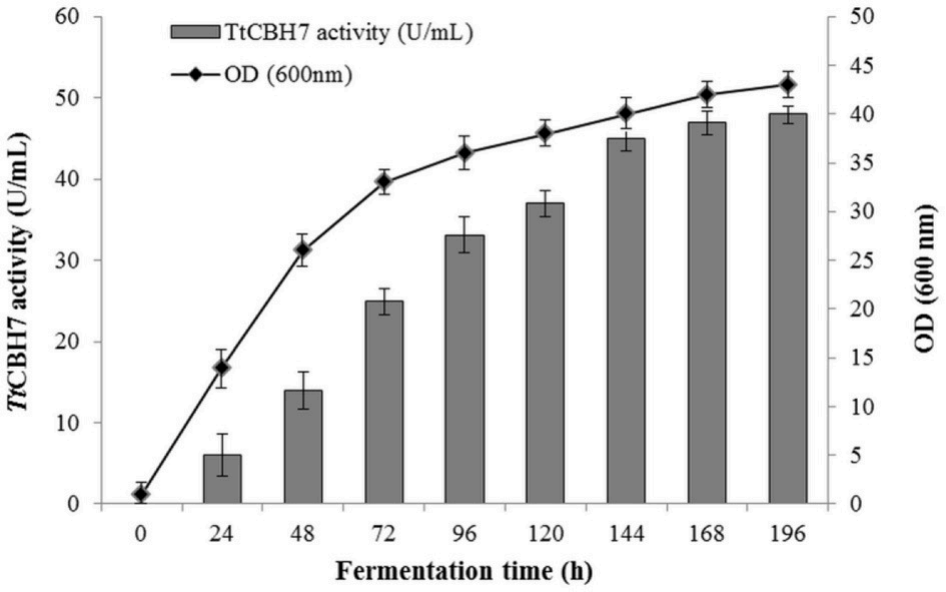
Time course of *Tt*CBH7 activity (*gray* bar) and biomass (*black* circle) production of the recombinant *P. pastoris* harboring the *cbh7* gene. The enzyme was expressed in culture broth by induction with 0.5% v/v methanol and addition of 20 g/L ammonium sulfate and its activity was measured with Avicel as substrate.

Protein expression of *Tt*CBH6 and *Tt*CBH7 was first evaluated in small scale shake flask cultures, and the clone that exhibited the highest activity against Avicel 5% w/v was chosen for the production of the recombinant enzymes and further characterization studies. *Tt*CBH6 was subsequently produced in high cell-density cultivation in bioreactor using the basal salts medium supplemented with PTM_1_ trace salts, according to Invitrogen, *Pichia* Fermentation Process Guidelines, reaching a maximum level of enzyme expression equal to 0.65 U/mL (activity against Avicel for varying time points shown at Figure 1). As methanol was used as carbon source, there was an increase in cell-density during the fed batch phase. At the end of the fermentation, the dry weight of cells reached 151.5 g/L and the total amount of crude extracellular protein obtained was 0.72 g/L. The production of *Tt*CBH7 was tested in 1 L shake flasks, in BMGY medium for 18–24 h. After examination of the CBH activity, no efficient yield of recombinant protein was achieved. The major factor causing this problem was primarily the proteolytic degradation of enzymes produced, which hampered the yield. Proteolysis led to low full-length recombinant protein levels and active products that were smaller than the full-length protein. Degraded proteins ran as a “smear” at SDS-PAGE (Figure S2). As a result, in spite of high protein amounts measured at the culture medium, only a small proportion was biologically active. In order to eliminate proteolysis and achieve higher production levels of homogenous and stable enzyme, several strategies were followed, and a series of different parameters were evaluated, such as the reduction of incubation temperature, the influence of initial pH, ammonium sulfate and methanol concentration and agitation. Of all the above parameters, it was found that when the initial concentration of ammonium sulfate in the culture medium was Two-fold higher than the one usually used (10 g/L, as suggested by EasySelect™ *Pichia* Expression Kit protocol), the protein appeared full length sized and homogenous. Activity on Avicel 5% w/v could be first detected in the medium 24 h after inoculation and peaked at 168 h with a titer of 47 U/ml (Figure 2).

**Figure 3:**
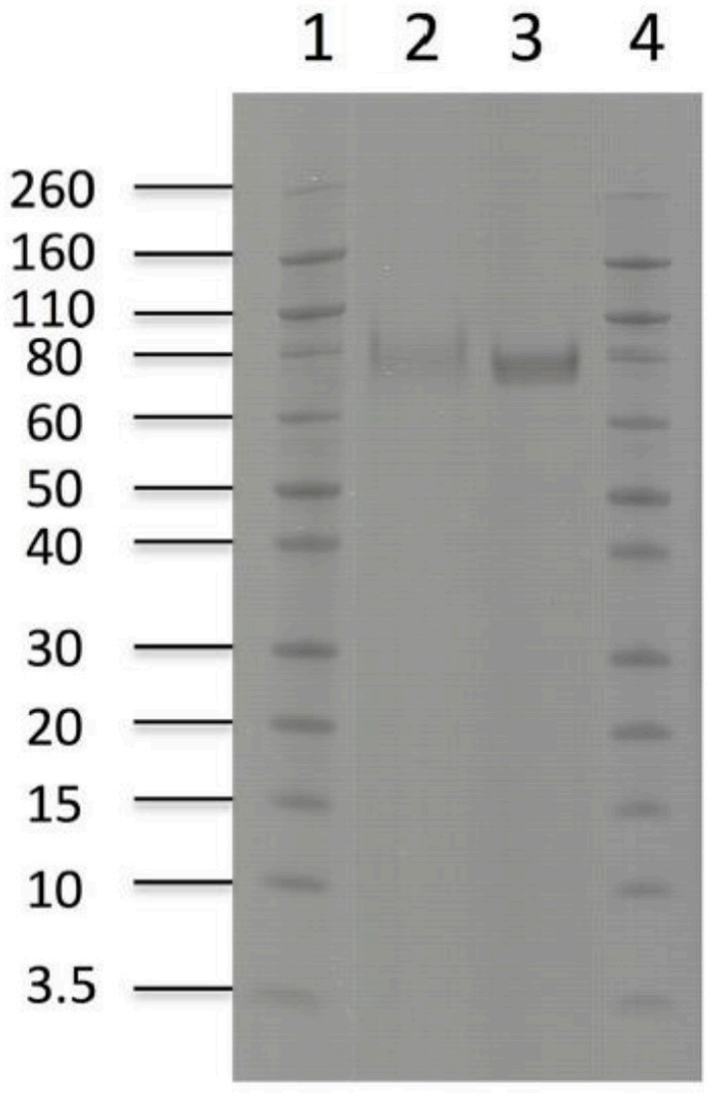
SDS-PAGE of enzymes used in hydrolysis experiments. *Lanes1,4*: Novex® sharp pre-stained protein marker, *Lane 2*: *Tt*CBH7, *Lane 3*: *Tt*CBH6.

Purification of the enzymes with immobilized metal ion affinity chromatography (IMAC) and removal of background impurities from the fermentation broth resulted in 294 mg of pure *Tt*CBH6 and 39.5 mg of pure *Tt*CBH7 per L of culture supernatant. The molecular weight of *Tt*CBH6 and *Tt*CBH7 was estimated to be ca. 75 and 78 kDa, respectively (Figure 3), which appears to be significantly higher than the predicted values using the ProtParam tool of ExPASY, even after considering the presence of the myc epitope and the polyhistidine tag. This observation might be explained by the existence of *N-* and *O-*glycosylation post-translational modifications. Indeed, 1 *Asn-Xaa-Ser/Thr* sequon and 44 *Ser-Thr* residues were predicted in *Tt*CBH6 sequence by using the NetNGlyc 1.0 server (http://www.cbs.dtu.dk/services/NetNGlyc/) and the NetOGlyc 3.1 server (http://www.cbs.dtu.dk/services/NetOGlyc/), while 1 *Asn-Xaa-Ser/Thr* sequon and 23 *Ser-Thr* residues were predicted in *Tt*CBH7 sequence.

The purified *Tt*CBH6 and *Tt*CBH7 were assayed for their activity toward different substrates. *Tt*CBH6 showed a specific activity of 1.63 ± 0.05 U/mg on Avicel 5% w/v, 1.19 ± 0.12 U/mg on β-glucan 0.1% w/v and 0.25 ± 0.09 U/mg for CMC 1% w/v. No activity of *Tt*CBH6 on pNP-substituted substrates was detected, while it had a low activity on PASC (1.10 ± 0.23 U/mg). *Tt*CBH7 was preferentially active toward PASC 0.5% w/v, exhibiting an activity of 3.21 ± 0.24 U/mg, followed by Avicel 5% w/v (2.81 ± 0.13 U/mg), while on CMC 1% w/v the specific activity was lower and reached 0.21 ± 0.11 U/mg. The activity of *Tt*CBH7 was also tested on pNP-β-cellobioside 5 mM (0.13 ± 0.08 U/mg) and pNP-β-lactopyranoside 5 mM (2.25 ± 0.05 U/mg). The optimal temperature activity of *Tt*CBH6 was observed at 60°C, losing rapidly its activity for temperatures over 70°C. The enzyme remained fairly stable up to 55°C after preincubation for 24 h in 0.1 M phosphate-citrate buffer pH 5.0 and exhibited half-life of 16.02 h at 60°C. The optimum temperature activity of *Tt*CBH7 was also observed at 60°C. The enzyme remained stable up to 50°C, after preincubation for 24 h and exhibited half-life of 18.1 h at 55°C and 9.41 h at 60°C, respectively. Both enzymes presented the highest activity levels at pH 5.0, while the activity dropped rapidly for pH less than 4 or higher than 6. *Tt*CBH6 and *Tt*CBH7 were found remarkably stable in the pH range 3–11 after 24 h retaining their initial activity.

**Figure 4:**
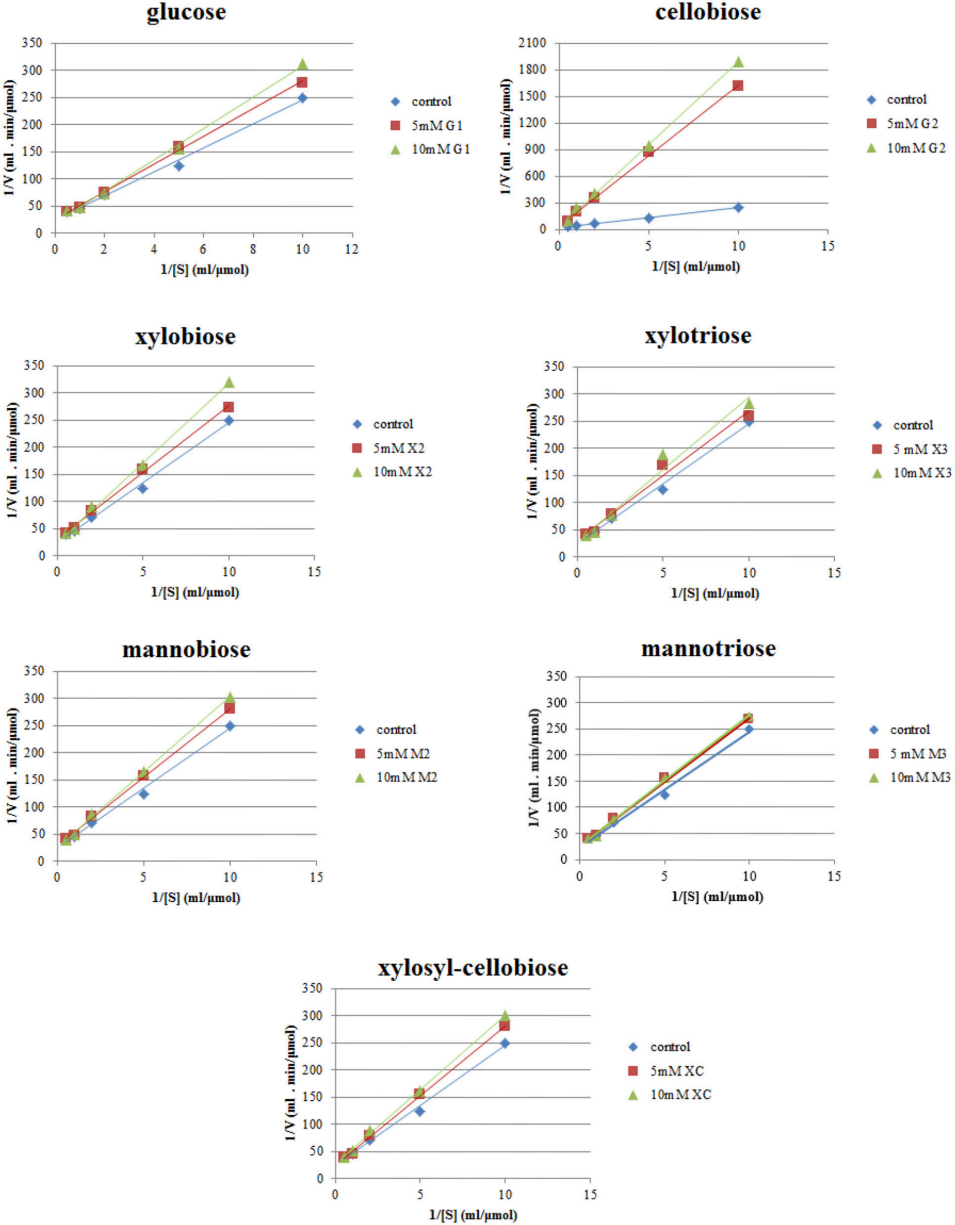
Inhibition studies of *Tt*CBH7 by glucan, xylan, and mannan mono- and oligo-saccharides. Absorbance at 355 nm was measured after 30 min incubation with MUG2 at concentrations ranging from 0.1 to 2 mM, at 45°C, pH 5.0.

Lineweaver–Burk plots of the *Tt*CBH6 and *Tt*CBH7 activities at different substrate concentrations of MUG2 in the absence and presence of different concentrations of inhibitors are shown in Figure 4. The inhibitory effect of sugars on *Tt*CBH7, when observed, indicated a competitive mode of inhibition, based on the intersection of the lines on the Y-axis, while no inhibitory effect on *Tt*CBH6 was observed for the inhibitors tested in concentrations of 5 and 10 mM (plots not shown). As depicted in Table 3, the K_*m*_ for MUG2 was 0.90 mM upon the absence of any inhibitor, while the ${\text{K}}_{m}^{app}$ increased to 1.58 and 6.54 mM in the presence of 10 mM glucose and 10 mM cellobiose, respectively, indicating that cellobiose is a strong inhibitor for *Tt*CBH7. The ${\text{K}}_{m}^{app}$ was 1.17 and 1.04 for 10 mM xylobiose and 10 mM mannobiose, while the respective trioses (xylotriose and mannotriose) had a very weak inhibitory effect on the enzyme activity. The ${\text{K}}_{m}^{app}$ values tend to increase following the inhibitors concentration increase, revealing that the competitive mode of inhibition.

**Table 3:** Enzyme kinetic parameters for glucan, xylan, and mannan mono- and oligo-saccharide inhibition of *Tt*CBH6 and *Tt*CBH7 using MUG2 as a substrate.

		***Tt*****CBH7**			***Tt*****CBH6**	
		**V*_*max*_* (μM/min)**	**K*_*m*_*/K*_*m*_^*app*^* (μM)**	**K*_*i*_* (mM)**	**V*_*max*_* (μM/min)**	**K*_*m*_*/K*_*m*_^*app*^* (μM)**
MUG2		41 × 10^∧^3	0.90		33 × 10^∧^3	0.42
Cellobiose	5 mM	25 × 10^∧^3	4.04	1.43	33 × 10^∧^3	0.40
	10 mM	35 × 10^∧^3	6.54	1.59	34 × 10^∧^3	0.45
Glucose	5 mM	39 × 10^∧^3	1.00		31 × 10^∧^3	0.39
	10 mM	54 × 10^∧^3	1.58	13.23	33 × 10^∧^3	0.41
Xylotriose	5 mM	32 × 10^∧^3	0.77		32 × 10^∧^3	0.42
	10 mM	36 × 10^∧^3	0.95		28 × 10^∧^3	0.46
Xylobiose	5 mM	33 × 10^∧^3	0.81		30 × 10^∧^3	0.44
	10 mM	40 × 10^∧^3	1.17	33.3	32 × 10^∧^3	0.41
Mannotriose	5 mM	36 × 10^∧^3	0.88		30 × 10^∧^3	0.41
	10 mM	39 × 10^∧^3	0.98		34 × 10^∧^3	0.44
Mannobiose	5 mM	36 × 10^∧^3	0.91		31 × 10^∧^3	0.38
	10 mM	38 × 10^∧^3	1.04		29 × 10^∧^3	0.35
Xylosyl-Cellobiose	5 mM	37 × 10^∧^3	0.99		30 × 10^∧^3	0.43
	10 mM	39 × 10^∧^3	1.01		33 × 10^∧^3	0.48

*There was no inhibition observed for TtCBH6 upon the addition of 5 and 10 mM concentration of different inhibitors. K_m_-value was calculated from the reaction in the absence of inhibitors*.

### Hydrolysis of natural substrates with commercial cellulases

Four different commercial enzymes (two CBHs and two EGs) representing the main cellulolytic activities were used for the design of a multi-component mixture and were tested against two organosolv pretreated lignocellulolytic substrates (spruce and birch) for the release of cellobiose. To determine the CBHI:EG5 ratio that gives the maximal % cellobiose yield, preliminary experiments with different combinations of CBHI and EG5 were conducted, using an enzyme loading of 25 mg/g substrate and incubating the reaction for 48 h. The results, as depicted in Table 4 showed that a relative abundance of CBH7 to EG5 equal to 60:40–70:30 (for spruce) and 50:50–60:40 (for birch) is required in order to maximize the cellobiose yield, corresponding to 31 and 38% of glucan conversion for spruce and birch, respectively. To evaluate the effect of EG12 on the % cellobiose yield, the enzyme was added at 3% of the total mixture and the results showed an increase of ~7% in the hydrolysis levels, yielding 36.7% conversion rates for spruce and 42.1% for birch. According to these results, the limits of the relative abundances of the enzymes for the experimental design **#1** were chosen. Table 5 shows the model prediction and the experimental results for % cellobiose yields using the four commercial enzymes, where cellobiose is expressed as a percentage of the total glucan content of the substrate*s*. The relative proportions of the enzymes were independently optimized for each substrate for 24 and 48 h.

**Table 4:** Preliminary experiments with various CBHI:EG5 ratios.

	**Birch**		**Spruce**	
**CBH7:EG5 ratio**	**Cellobiose mg/mL**	**Cellobiose yield (%) (*mg/g glucan*)**	**Cellobiose mg/mL**	**Cellobiose yield (%) (*mg/g glucan*)**
1:100	2.87 ± 0.12	19.0 (*190*)	1.96 ± 0.04	13.2 (*132*)
40:60	5.43 ± 0.89	35.9 (*359*)	4.19 ± 0.31	28.2 (*282*)
50:50	**5.74** ± 0.67	**38.0 (*380*)**	4.57 ± 0.23	30.7 (*307*)
60:40	**5.78** ± 0.51	**38.3 (*383*)**	**4.62** ± 0.61	**31.1 (*311*)**
70:30	5.48 ± 0.55	36.3 (*363*)	**4.65** ± 0.50	**31.3 (*313*)**
80:20	5.08 ± 0.46	33.6 (*336*)	4.51 ± 0.11	30.3 (*303*)
90:10	4.64 ± 0.21	30.7 (*307*)	4.41 ± 0.14	29.7 (*297*)
100:1	3.57 ± 0.39	23.7 (*237*)	3.82 ± 0.22	25.7 (*257*)

*The reactions were performed with 25 mg total enzyme/g substrate and incubated for 48 h. The results are expressed as a percentage of the conversion of the total glucan content of the substrates. Bold values indicate the enzyme combinations giving the maximal hydrolysis yields*.

**Table 5:** Model prediction values and experimental results of the optimized mixtures and final % cellobiose yields, after 24 and 48 h of reaction for #1 and #2 experimental design.

**#1**	**Substrate**	**Optimal enzyme proportions (%)**				**Cellobiose yield (%)**	
		**CBH7**	**CBH6**	**EG5**	**EG7**	**Mod. Pr**.	**Exp. data**
24 h	Spruce	66.6	5.0	23.3	5.0	22.7	22.3 ± 1.7
	Birch	58.5	6.5	30.0	5.0	26.4	27.1 ± 2.3
48 h	Spruce	50.0	12.0	27.0	11.0	37.7	36.2 ± 0.5
	Birch	50.0	5.0	30.0	15.0	43.8	40.2 ± 1.2
**#2**	**Substrate**	**Optimal enzyme proportions (%)**				**Cellobiose yield (%)**	
		**CBH7**	**EG5**	**EG9**	**EG6**	**Mod. Pr**.	**Exp. data**
48 h	Spruce	64.9	21.6	4.3	9.2	42.5	40.0 ± 2.1
	Birch	69.3	20.0	9.7	1.0	41.6	42.7 ± 1.1

*In experimental design #2, CBH6 and EG7 were kept constant and equal with those values that gave the maximal cellobiose yield in design #1. The % cellobiose yields have been calculated on the basis of total glucan content of initial substrates*.

### Hydrolysis of organosolv pretreated spruce

**Figure 5:**
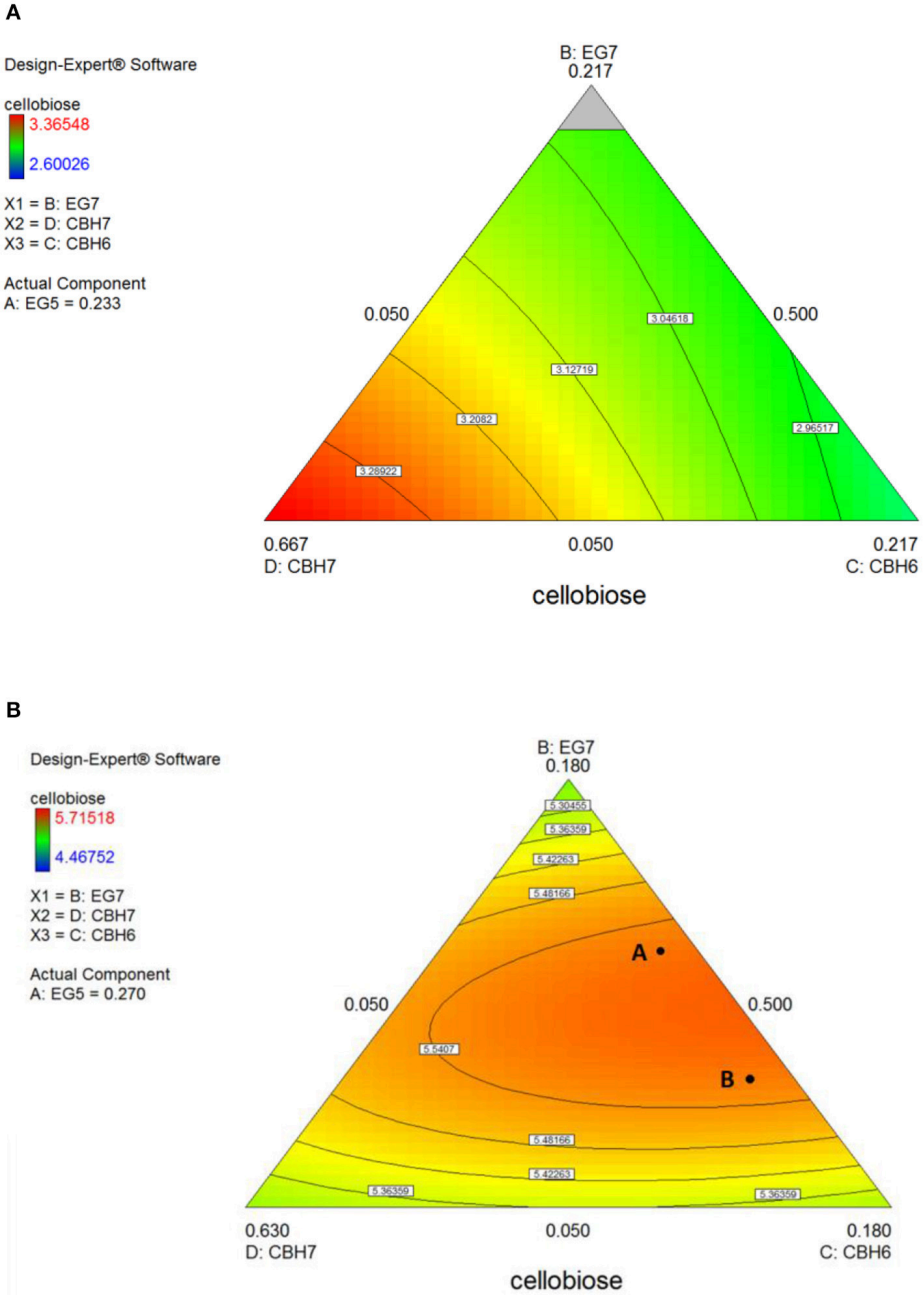
Ternary plots for the experimental design **#1**, showing predicted final total cellobiose concentration (mg/mL) from *spruce* hydrolysis for 24 h **(A)** and 48 h **(B)**, as a function of three out of four enzymes (CBH7, CBH6, EG7) content.

The maximum concentration of cellobiose released from the hydrolysis of *spruce* was calculated using the quadratic model (*p* = 0.0025, *R*^2^ = 0.8655 for 24 h and *p* = 0.0015, *R*^2^ = 0.8796 for 48 h, Figure S3 and Table S7) and reached 3.37 and 5.6 mg/mL after 24 and 48 h incubation, respectively (Table S5). After 24 h, the % cellobiose yield corresponded to 22.7 ± 1.7% of the initial glucan content of the substrate and was achieved with high levels of CBH7 (66.6%) and EG5 (23.3%; Table 5). The experimental data with the optimal ternary mixture were very close to the predicted ones, resulting in 22.3% hydrolysis. As illustrated in Figure 5A and Figure S4, a decrease in CBH7 proportion resulted in steep drop in cellobiose concentration, even if EG7 and CBH6 levels are high, indicating the key role of this enzyme for the reaction. EG5 is also depicted as an important enzyme for the glucan conversion to cellobiose. With the relative proportion of CBH7 constant and equal with 66.6%, the value that corresponds to that of the optimized mixture, the hydrolysis is mostly dependent on the levels of EG5, as depicted in Figure S4Aii. When being together, CBH7 and EG5 comprise 89.9% of the total enzyme mixture and they can partially compensate each other, as shown at Figures S4Aiii–iv and has also been verified by the preliminary results mentioned above.

**Figure 6:**
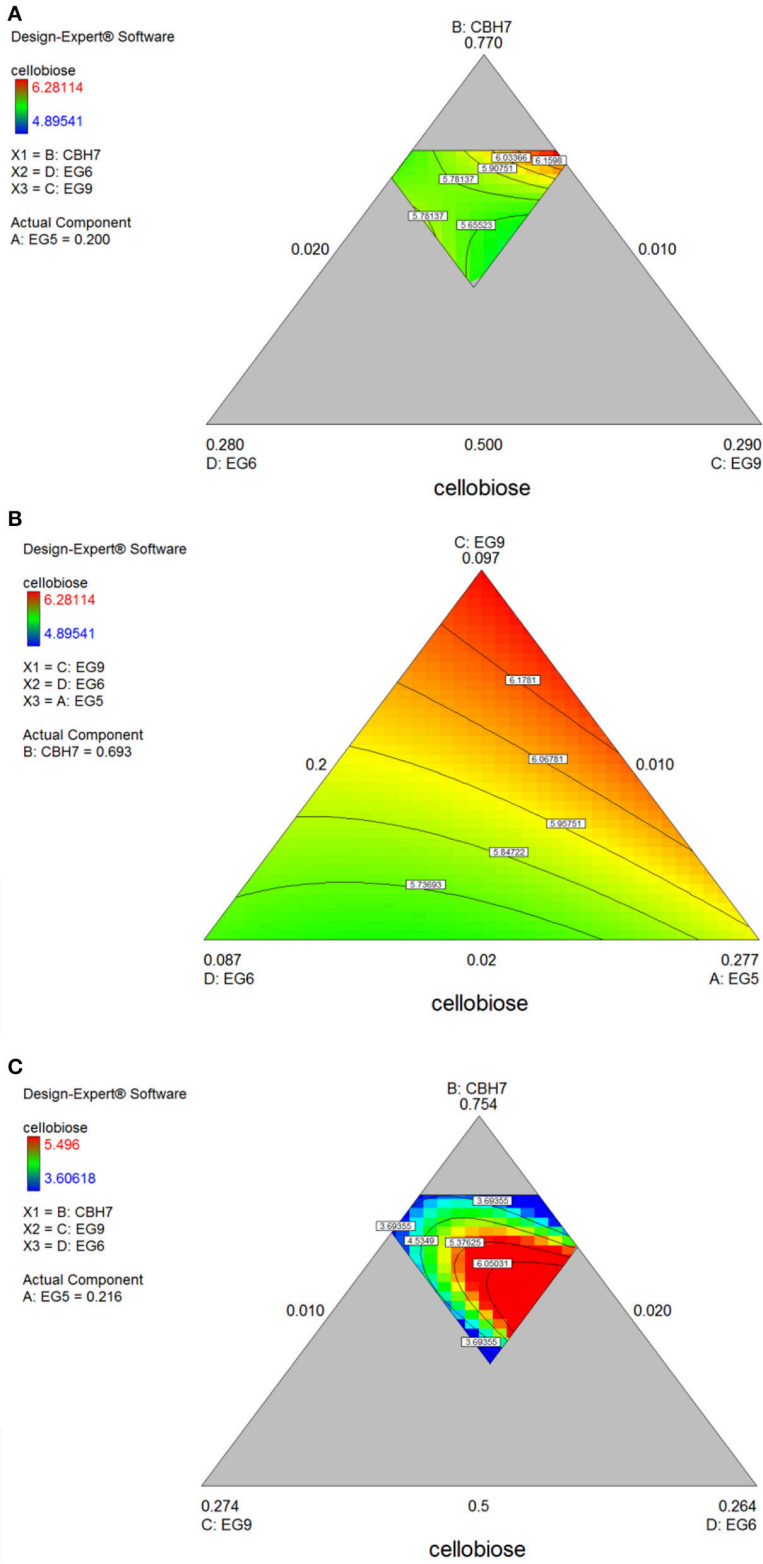
Ternary plots for the experimental design **#2** showing predicted final total cellobiose concentration (mg/mL) from *spruce* **(A,B)** and *birch* **(C)** hydrolysis, as a function of three out of four enzymes (CBH7, EG6, EG9 or EG9, EG6, EG5) content.

After 48 h of hydrolysis, optimal % cellobiose yield was predicted to be 37.7% with a ternary mixture of 50% CBH7, 12% CBH6, 27% EG5, and 11% EG7. CBH6 and EG7 appear in higher proportions to the composition of the optimal mixture than they did for 24 h hydrolysis. Experimental values showed a slightly decreased yield (36.2 ± 0.5%) in comparison to the predicted one. From the ternary plot of Figure 5B it can be concluded that the contribution of CBH6 and EG7 is more important in this stage of hydrolysis than that at 24 h, as when moving from point **A** to point **B**, CBH6 can compensate for EG7 and maintain high cellobiose yields, while EG5 is constant and equal with the proportion of the optimal cocktail. From Figure S4Bii it is also obvious that, when CBH7 comprises 50% of the final mixture and the proportions of EG5 decrease below a certain level, the hydrolysis rate drops. In contrast, as shown in Figure S4Biii, CBH7/EG7 together can partially compensate for the low amount of EG5, so the high cellobiose concentration is conserved. In experimental design **#2**, CBH7 and EG5 comprise ~85% of the optimal mixture of the processive enzymes, resulting to a cellobiose production of 6.3 mg/mL that corresponds to 42.5% substrate conversion (Table S6). The ternary mixture, as calculated by the special cubic model (*p* = 0.0111, *R*^2^ = 0.9409; Table S8) was 64.9% CBH7, 21.6% EG5, 4.3% EG9, and 9.2% EG6. Apart from CBH7 and EG5, EG6 also stands necessary for achieving high yields, as depicted in Figure 6C and Figure S6B.

### Hydrolysis of organosolv pretreated birch

**Figure 7:**
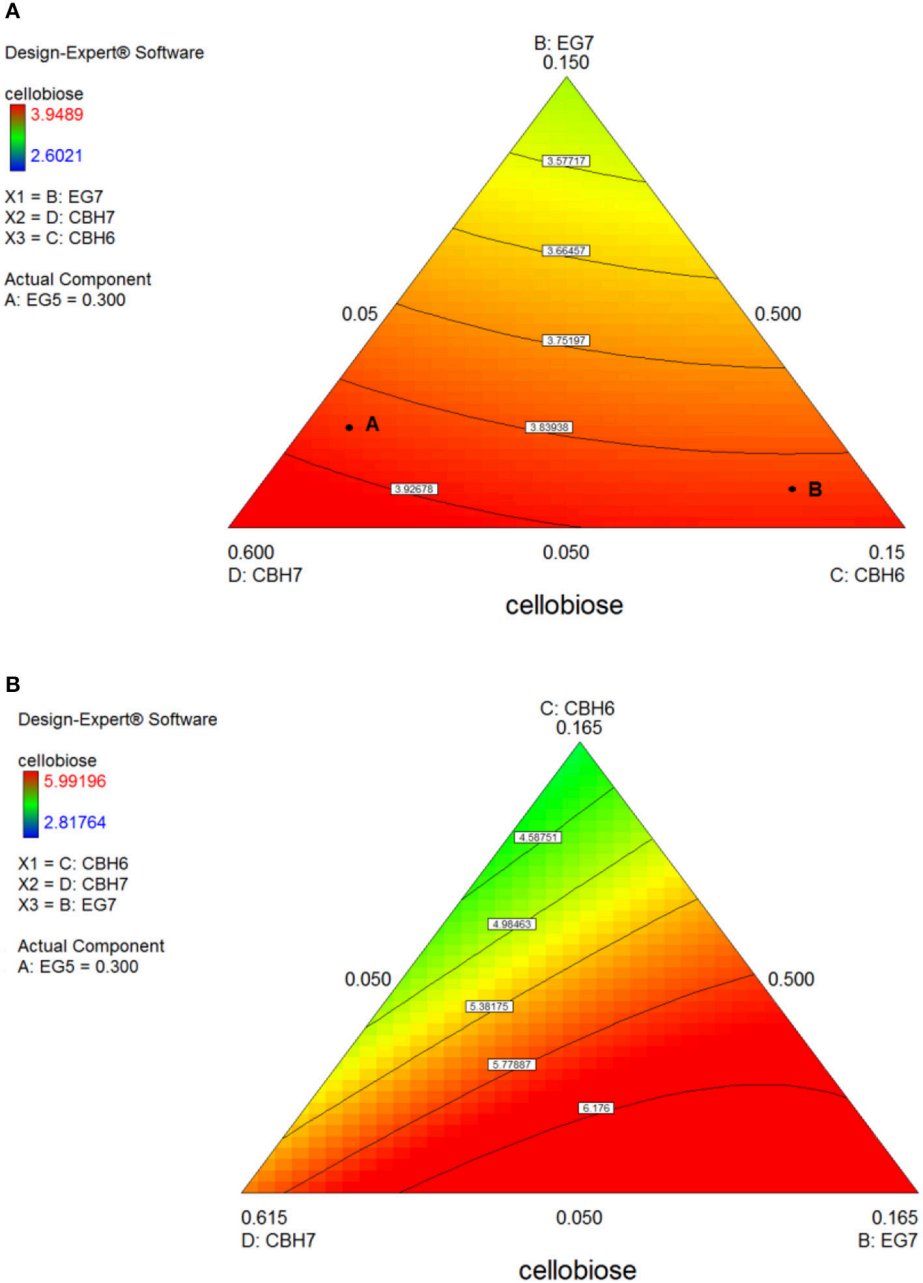
Ternary plots for the experimental design **#1**, showing predicted final total cellobiose concentration (mg/mL) from *birch* hydrolysis for 24 h **(A)** and 48 h **(B)**, as a function of three out of four enzymes (CBH7, CBH6, EG7) content.

After 24 h of reaction, the highest product formation was 3.98 mg/mL which corresponded to 26.37 % of glucan conversion (Table S4) and was achieved with a ternary mixture of 58.5% CBH7, 6.5% CBH6, 30% EG5, and 5% EG7, as determined by the quadratic model (*p* < 0.0001, *R*^2^ = 0.9484, Figure S3 and Table S7). Experimental values were close to the predicted ones (27.1 % ± 2.3). The ternary plot of Figure 7A and Figure S5Ai shows that when the proportion of EG5 is equal to that one of the optimized cocktail (30%), an increase of CBH7 and decrease of CBH6 or *vice versa*, within the limits of the experimental design, practically has no effect on the hydrolysis rate which remains relatively high, for example when moving from point A to B. This is indicative of the pivotal importance of EG5 for the cellobiose production from birch, which is also apparent in Figures S5Aii–iv. Aside from EG5, the activity of CBH7 has a great impact, as it consists 58.5% of the total enzyme mixture that maximizes the substrate conversion.

The maximum yield of cellobiose released from the 48-h hydrolysis of birch was 6.6 mg/mL and corresponded to 43.8%, according to the quadratic model prediction (*p* = 0.0168, *R*^2^ = 0.7932, Figure S3). It was achieved with an enzyme combination of 50% CBH7, 5% CBH6, 30% EG5, and 15% EG7. The experimental values were slightly lower (40.2% ± 1.2) in comparison to the predicted one. The pivotal importance of EG5 and CBH7 for the maximal cellobiose yields, similarly to the results after 24 h of reaction, is shown at Figure 7B and Figures S5Bi-iv, with the difference that EG7 contributes in higher proportions and can partially compensate for CBH7. From the Figure S5Bii, it is profound that when EG5 is in its lower limit proportion, the hydrolysis rate is very low. In experimental design **#2**, similarly to the results from spruce hydrolysis, CBH7 and EG5 comprise ~90% of the optimal mixture of the processive enzymes, resulting to a cellobiose production of 6.28 mg/mL that corresponds to 41.64% substrate conversion (Table S6). The ternary mixture, as calculated by the special cubic model (*p* = 0.0094, *R*^2^ = 0.8189) was 69.3% CBH7, 20% EG5, 9.7% EG9, and 1% EG6 (Table S8). Apart from CBH7 and EG5, the requirement of EG9 to high yields product formation is also highlighted, as shown in Figures 6A,B and Figure S6A.

### Evaluation of *Tt*CBH6 and *Tt*CBH7 in ternary mixtures

After identifying the optimal mixture that maximizes cellobiose production using commercial enzymes, *Tt*CBH6 and *Tt*CBH7 were used to replace CBH6 and CBH7 in optimized combinations in order to evaluate the performance of these *in-house* produced enzymes and compare with that of commercially available CBHs. The results showed that the hydrolysis rates were slightly lower when compared to the optimized mixtures that contain the commercial CBHs, achieving 32 ± 1.1 and 35.2 ± 1.8% of spruce and birch conversion, respectively, with the #1 enzyme mixture (Table 6). With #2 enzyme mixture, the yields reached 36 ± 0.9% for spruce and 35.2 ± 2.1% for birch.

**Table 6:** Experimental values of % cellobiose yields after 24 and 48 h of reaction, using the optimized ternary mixtures identified by #1 and #2 designs, after replacement of commercial CBH6 and CBH7 with *Tt*CBH6 and *Tt*CBH7 that were produced and characterized in the present study.

**#1**	**Substrate**	**Optimal enzyme proportions (%)**				**Cellobiose yield (%) (*mg/g glucan*)**
		***Tt*CBH7**	***Tt*CBH6**	**EG5**	**EG7**	
*24 h*	Spruce	66.6	5.0	23.3	5.0	18.4 ± 2.7 (*184*)
	Birch	58.5	6.5	30.0	5.0	28.1 ± 0.3 (*281*)
*48 h*	Spruce	50.0	12.0	27.0	11.0	32.0 ± 1.1 (*320*)
	Birch	50.0	5.0	30.0	15.0	35.2 ± 1.8 (*352*)
**#2**	**Substrate**	**Optimal enzyme proportions (%)**				**Cellobiose yield (%) (*****mg/g glucan*****)**
		***Tt*****CBH7**	**EG5**	**EG9**	**EG6**	
*48 h*	Spruce	64.9	21.6	4.3	9.2	36.0 ± 0.9 (*360*)
	Birch	69.3	20.0	9.7	1.0	35.2 ± 2.1 (*352*)

*The % cellobiose yields have been calculated on the basis of total glucan content of initial substrates*.

## Discussion

Large-scale production of cellobiose and other sugars can occur via enzymatic or acid-catalyzed hydrolysis, or a combination of these processes (Lim and Lee, 2013). Acid-catalyzed processes have been extensively reported throughout the literature, either as an initial pretreatment step to facilitate the subsequent enzymatic saccharification yields, either by using solid acid catalysts in heterogeneous catalysis approaches (Lim and Lee, 2013; Vilcocq et al., 2014). Apart from the low yield and the formation of toxic by-products, such as furfural and 5-hydroxymethylfurfural (HMF), the use and handling of concentrated acids create additional problems. Enzymatic hydrolysis processes offer numerous benefits, as they are typically milder, environmentally friendly and are carried out under lower pressure and at ambient temperature while producing less by-products. Until now, the major bottleneck that could possibly hamper the wide use of enzymes is the slow conversion rate after the initial hydrolysis stage, which is attributed to the biomass recalcitrance (Auxenfans et al., 2017), and their high-production cost. As a result, efforts are made to improve the enzyme performance and produce efficient biocatalysts, toward a reduced enzyme consumption and, thus, to increase the overall sustainability of the process. The main enzymes for the cellobiose production are CBHs and processive EGs. The latter enzymes have gained much attention recently, after some microorganisms were reported to be able to degrade cellulose by using only EGs with both endo- and exo-activity that act in processive manner, while they completely lack exoglucanases from their genome (Watson et al., 2009; Zhu et al., 2013). These findings raised the interest and questioned the role of the processive enzymes for the degradation of cellulose and their interactions with CBHs. In this context, we report here the heterologous expression and characterization of two CBHs from *T. thermophila* in the methylotrophic yeast *P. pastoris*. The two enzymes were tested in optimized enzyme mixtures together with processive endoglucanases for their ability to release high amounts of cellobiose. *T. thermophila* is a filamentous ligno-cellulolytic ascomycete with a wide range of synonyms over the history of its classification and distinction of sexual states (previously known as *Sporotrichum thermophilum and Myceliophthora thermophila*; Marin-Felix et al., 2015). A repertoire of enzymes, including GHs, oxidoreductases and esterases from this fungus have been widely studied (Topakas et al., 2012; Charavgi et al., 2013; Karnaouri et al., 2014, 2017; Zerva et al., 2016).

Even though numerous studies report the expression of CBHs in the methylotrophic yeast *P. pastoris* and other yeasts (Boer et al., 2000; Igarashi et al., 2012; den Haan et al., 2013; Fang and Xia, 2015; Woon et al., 2017), their use in scale-up processes is hampered due to their weak enzymatic activities together with the low production yields from the host cells. Weak enzymatic activities can be related to the heterogeneity of glycosylation patterns promoted by yeasts cells that, though in several cases increases the thermostability of the enzymes and the resistance to proteolytic attack (Amore et al., 2017), it may interact with the enzyme active site (Cereghino and Cregg, 2000; Mattanovich et al., 2012). Heterogeneity in glycosylation is a result of differences in oligosaccharide-protein populations in terms of the type, length, and identity of the oligosaccharides added, resulting in protein products whose micro-properties, such as the isoelectric point, vary slightly. In our study, the molecular weight of both *Tt*CBH6 and *Tt*CBH7 was significantly higher than the theoretical one, which was attributed to the existence of *N-* and *O-*glycosylation post-translational modifications. Aside from glycosylation, many processive enzymes, especially CBHs, exhibit N-terminal modifications, such as the formation of a pyroglutamate residue, which is necessary for their activity; this modification is lacking in *P. pastoris* and other yeast systems for heterologous expression (Dana et al., 2014). Low production yields from the heterologous expression cells can also occur due to extensive proteolysis.

Proteolytic degradation has been a perpetual problem during the production of recombinant enzymes, especially when yeasts, such as *P. pastoris*, are employed (Gimenez et al., 2000). Proteolysis is susceptible to amplify over the fermentation induction period, following the reduction of the number of viable cells in the culture and is related mainly to the vacuole proteases (van den Hazel et al., 1996). Though several studies have been focused on the proteolytic degradation of the extracellular recombinant proteins, there is no in-depth analysis on the parameters that could promote proteolysis or the type of the proteases implied. *Addition of ammonium ions* in the form of ammonium sulfate has been recommended in the literature as a possible strategy to reduce proteolysis. Tsujikawa et al. (1996), observed a 10-fold reduction of proteolysis by supplementing medium with ammonium ions. In another study, the addition of 20 g/L (NH_4_)_2_SO_4_ to a culture medium has been reported not only to increase the amount of glucoamylase produced but also for maintaining glucoamylase activity at a high level during the fermentation (Kobayashi and Nakamura, 2003). It was suggested that limited proteolysis can be attributed to the fact that the solubility of the proteases decreases upon the addition of (NH_4_)_2_SO_4_ by sulfate conjugation, leading to decrease of the total proteolytic activity in the culture supernatant. País-Chanfrau et al. (2004) also reported an increased expression of recombinant mini-proinsulin in *P. pastoris* in bioreactors, achieved by, among others, periodical addition of ammonium sulfate. In this study, we describe the successful expression of *Tt*CBH7 in *P. pastoris*, under high osmotic pressure and increased salinity conditions. Two-fold increase of the ammonium sulfate in the culture medium resulted in the production of full-length product, as shown by the higher protein purity on SDS-PAGE gel.

*Tt*CBH6 and *Tt*CBH7 were shown to retain 100% of activity after 24 h incubation at 55 and 50°C, respectively, while they both showed activity on β-glucan. *Tt*CBH7 was active on a wide range of substrates, including crystalline, amorphous, and pNP-substituted substrates, as described above, whereas *Tt*CBH6 showed selectivity, with no detectable activity on pNP-substrates. However, *Tt*CBH6 exhibited a higher affinity for MUG2 in comparison to *Tt*CBH7, as depicted by the K_*m*_ values, which has also been reported in the literature (Badino et al., 2017). It is related to the distinct catalytic properties and active site configuration observed in CBHs belonging to different families, with the GH6 CBHs to present weaker interactions with the cellulose substrate, which leads to higher association-dissociation rates and, consequently, higher catalytic rates. Both CBHs exhibit fairly high thermostability, which supports the potential application of these enzymes in the lignocellulosic materials degradation processes. Though CBHs have a major role to the conversion of the substrate to cellobiose, their activity is usually characterized as slow and susceptible to inhibition. The effect of glucan-, xylan-, and mannan-fragments have been widely studied as one of the main factors limiting the activity of CBHs, with cellobiose being the main compound that causes the enzyme end-product inhibition (Selig et al., 2008; Baumann et al., 2011). Depending on the pretreatment methods used, the type of the substrate and the pretreatment severity factor that is applied, variable amounts and different types of hemicelluloses may remain in the solid fraction of lignocellulosic materials. In hardwoods and agricultural plants, the main hemicellulosic structure is xylan, while softwoods are dominated by glucomannan. After enzymatic hydrolysis which usually takes place by employing a commercially available cellulolytic enzyme mixture which also contains side enzymatic activites acting on xylan and mannan structures, xylo- and manno-oligomers and monomers are released as hydrolysis products. These products, especially when found in increased concentrations, regularly cause inhibition of cellulases, with CBHs to be primarily affected (Qing et al., 2010). The structural similarity of xylobiose, mannobiose, and cellobiose may result in their competitive binding onto the active site of CBH forming a steric hindrance and, preventing the glucan chain to move into the tunnel, thus leading to a sharp decrease in their hydrolytic activity (Baumann et al., 2011). CBHs belonging to family 6 have been reported to be less susceptible to end product inhibition than those belonging to family 7 (Hele Teugjas and Väljamäe, 2013; Badino et al., 2017). In this study, no inhibitory effect of glucose, cellobiose, xylo- and manno-oligomers on *Tt*CBH6 was observed for inhibitor concentrations up to 10 mM, whereas *Tt*CBH7 was found to be strongly inhibited, especially by cellobiose.

Several strategies have been proposed with the aim to boost the enzymatic hydrolysis and increase cellobiose yields, including, among others, enzyme recycling by using multi-stage processes (Vanderghem et al., 2010, 2012) or product removal using membrane filtration (Gavlighi et al., 2013). The approach of the current study was to use *tailor-made* enzyme combinations and test their efficiency in glucan conversion to cellobiose. Commercially available cocktails have been optimized targeting hydrolysis of specific type of substrate. Moreover, when cellobiose production is the main target product, although it is possible to inhibit the β-glucosidase activity upon addition of an inhibitor such as D-gluconolactone (Reese et al., 1971; Dale et al., 1985), these cocktails also contain other hemicellulolytic activities, as mentioned above, resulting in production of oligomers that can act as inhibitors of cellulase activity, especially CBHs. Since different lignocellulosic biomass residues have heterogeneous properties by nature and consequently degradability, tailor-made sets of enzymes are required for conversion of each substrate. CBHs belonging to GH7 family are the major enzymes for the production of cellobiose, but they are susceptible to severe end-product inhibition; supplementation with processive EGs can help alleviating that problem, as the latter have a cleft-like active site with a looser structure than the tunnel-shaped binding region of CBHs which is not easy to be blocked by the inhibitors (Murphy et al., 2009; Watson et al., 2009). This is verified by the preliminary results of this study, where a combination of CBHI and EG5 in appropriate proportions can achieve up to Two-fold increase of hydrolysis rates when compared to CBHI or EG5 alone; these results are more profound in case of birch that has higher cellulose and lower lignin content than spruce.

Comparing the theoretical predicted and the experimental values for 24 and 48 h of hydrolysis (Table 4), it is obvious that the reaction rate is higher at the initial conditions but seems to decrease in later stages of the reaction. Numerous factors have been suggested to affect the hydrolysis yields and result in declined hydrolysis rate at the late stage, including high cellulose crystallinity that renders the substrate inaccessible to the enzymes, reduction of enzyme stability and non-specific adsorption of enzymes on the substrate, especially on lignin components (Chundawat et al., 2011). In this study, both lignocellulosic materials have undergone organosolv pretreatment with the use of ethanol as organic catalyst resulting in a high amount of cellulose together with low amount of lignin, thus minimizing the adsorption of cellulases and increasing their catalytic efficiency. *Organosolv* is typically applied as a pretreatment process step of lignocellulosic residues within the biorefinery and enables their fractionation through solubilization of hemicellulose and lignin fractions into the cooking liquor and production of a cellulose-rich solid stream highly susceptible to enzymatic hydrolysis (Sannigrahi et al., 2010; Nitsos et al., 2018). The hydrolysis rates were slightly higher when birch was used as a substrate, which could be possible attributed to the lower lignin content of birch (7.1%) compared to spruce (14.9%). It has been observed that the carbohydrate-acting enzymes and, more specifically, celullases possessing a CBM module can be adsorbed non-selectively onto lignin compounds of the substrate; this interaction possibly hampers their activity and leads to lower production yields.

The composition of optimal ternary mixtures for design **#1** show that CBH7 and EG5 together consist the main component of the reaction mixture producing the highest cellobiose yields; with CBH7 to be the prevailing component. As the incubation time increases from 24 to 48 h, the participation of EG7 and CBH6 becomes more profound for both spruce and birch. Possible factors that could explain this is either the end-product inhibition of CBH7 or the limited number of active sites as EG5 and CBH7 compete for the same sites and there is a depletion, so EG7, as an enzyme with a true endo-action, creates new chain ends for CBHs and EG5. *Exo-endo synergy* between CBHs and non-processive EGs, like EG7 is usually attributed to the ability of the latter to act on the middle-chain of cellulose molecule, thus creating two new chain ends, while the enzymes with a processive mode of action prefer to complex with the ends of the glucan chain (Jalak et al., 2012). Regarding the role of CBH6, this enzyme exhibits a greater impact during the later stages of hydrolysis, as shown by the results after 48 h of incubation. Although CBH II enzymes are regularly known to participate in the deconstruction of the crystalline cellulose areas, it has been proved that they can act in concert with EGs by removing amorphous cellulose and polishing the crystalline substrate regions so as to enable the attack of CBHI (Ganner et al., 2012), which is crucial in order to create new sites for the action of CBHI. Up to now, enzymes belonging to GH6 family are the only ones known that present an inverting catalytical mechanism and attack from the non-reducing end of cellulose chain (Cantarel et al., 2009). CBHI and CBHII enzymes exhibit exo/exo synergy, acting on the opposite ends of the glucan chain and using complementary modes of catalytical activity.

In experimental design **#2**, where all processive enzymes were used together with CBH6 and EG7, the conversion rate was lower that design **#1**, where only the four “core” enzymes (CBH7, CBH6, EG5, EG7) were used. In these reactions, CBH6 and EG7 proportions were kept constant and equal with those values that gave the maximal cellobiose yield in design **#1**. The ternary mixtures revealed that CBH7 stands as the most important enzyme among the processive activities, comprising 65–70% of the total cocktail of processive enzymes, for both spruce and birch, while the proportion of EG5 is 20%. The experimental values for hydrolysis rates after 48 h of reaction appear relatively close, but slightly higher than those determined in **#1**, despite the addition of processive enzymes from different GH families that would theoretically increase the cellobiose yields. This can be possibly attributed to the competition for the same binding sites or a phenomenon known as “traffic jam” which has been proposed to obstruct the activity of the CBHI enzymes (Igarashi et al., 2011). Replacement of commercial CBH6 and CBH7 in optimal ternary mixtures of design **#1** and **#2** with *Tt*CBH6 and *Tt*CBH7 produced in this study, led to relatively lower hydrolysis rates, but still a 35% of substrate conversion was achieved, which is rather high. This offers new perspectives for the use of *in-house* produced enzymes with specified and distinct activities to replace and/or supplement the commercial enzyme mixtures.

## Conclusion

The efficient cellobiose production from lignocellulosic materials requires enzymes that act on the reducing and non-reducing cellulose chains. *Tt*CBH6 and *Tt*CBH7 from *T. thermophila* are two CBHs that were heterologously produced in *P. pastoris* and fully characterized with the aim to be tested in optimized enzyme mixtures for cellobiose production from organosolv pretreated spruce and birch. The impact of a changing cocktail composition on the cellobiose yields was evaluated, leading to the development of tailor-made mixtures, customized for each substrate. Determination of the distinct role of the individual enzymes and their synergistic interactions revealed that CBH7 and EG5 are the key enzymes for the production of cellobiose at the early stage of the reaction, but other enzymes acting on the amorphous areas, such as EG7 and CBH6, are needed as the reaction proceeds. These enzymes contribute to the generation of new chain ends to promote the action of the processive enzymes. Replacement of commercial CBHs with *Tt*CBH6 and *Tt*CBH7 resulted in yields that, athough slightly lower, could still compete with the **#1** and **#2** optimized mixture performances, reaching 35% of substrate conversion to cellobiose. Processive EGs coupled with CBHs could alleviate the adverse effects of end-product inhibition of CBHs and, thus present a new perspective for the cellobiose production from lignocellulosic materials.

## Author contributions

AK: Carried out the experiments and wrote the manuscript; ET: Contributed in the molecular cloning and heterologous expression of the enzymes; LM: Performed the pretreatment and the compositional analysis of the forest materials; UR and PC: Participated in study conception, data interpretation and corrected the manuscript. All authors have read and approved the final manuscript.

### Conflict of interest statement

The authors declare that the research was conducted in the absence of any commercial or financial relationships that could be construed as a potential conflict of interest.
