# Supplementary material for: Fine-Tuned Enzymatic Hydrolysis of Organosolv Pretreated Forest Materials for the Efficient Production of Cellobiose
Source: Front Chem. 2018 Apr 19;6:128. doi: 10.3389/fchem.2018.00128 (PMC5917092; doi:10.3389/fchem.2018.00128)
Supplement: Supplementary file 1 [file DataSheet1.DOCX]

**“Fine-tuned enzymatic hydrolysis of organosolv pretreated forest materials for the efficient production of cellobiose”**

**Supplementary material**

**TABLES**

**Table S1.** Primers **(A)** and conditions **(B)** used for the amplification of *cbh6* gene through PCR (**#1**), removal of introns (**#2-4**) and final overlapping PCR (**#5**). Colored sequences represent the restriction sites of *ClaI* and *XbaI* enzymes (*red, purple*), as well as the complementary DNA fragments that allowed the hybridization reaction and the amplification of the generated “*fusion*” fragment by overlapping PCR (*blue: annealing, green: overhang*).

**(A)**

|  | primer sequence | |
| --- | --- | --- |
| *Tt*CBH6_66729**F** (21 bp) | 5' **GAC TCA ATG CGG CGG TAA CGG** 3' | |
|  |  | |
| *Tt*CBH6_66729**R** (29 bp) | 5' **CGT CTA GAA AGG GCG GGT TGG CGT TGG TG** 3' | |
|  | *XbaI* | |
| *Tt*CBH6_66729**e2F** (69 bp) | | 5' **GCA TCG ATG GCC CCC GTC ATT GAG GAG CGC**  *ClaI*  **CAG AAC TGC GGC GCT GTG TGG ACT CAA TGC GGC GGT AAC** 3' |
|  | | primer sequence (OE-PCR) |
| *Tt*CBH6_66729**e2R**  (38 bp) | | 5' **GTA GAC GAC GAG TTG GGC AGC ATA GGG AGG ATT GGC AC** 3' |
| *Tt*CBH6_66729**e3F** (21 bp) | | 5' **CCC AAC TCG TCG TCT ACG ACC** 3' |
| *Tt*CBH6_66729**e3R** (40 bp) | | 5' **CAC CCC ACT GTT GTT GGC CGG TAG GTT GTT TGC CGT TGC G** 3' |
| *Tt*CBH6_66729**e4F** (22 bp) | | 5' **GCC AAC AAC AGT GGG GTG ACT G** 3' |

**(B)**

| **PCR** | **Primers** | **Fragment targeted** | **Conditions** |
| --- | --- | --- | --- |
| **#1** | **EF - ER** | 1614 bp | annealing: 58^o^C, 10s extension: 70^o^C/ 32s, 30 cycles |
| **#2** | **Ee2F – Ee2R** | 550 bp | annealing: 55^o^C, 10s extension: 68^o^C/ 12s, 30 cycles |
| **#3** | **Ee3F – Ee3R** | 585 bp | annealing: 55^o^C, 10s extension: 68^o^C/ 15s, 30 cycles |
| **#4** | **Ee4F - ER** | 263 bp | annealing: 57^o^C, 10s extension: 70^o^C/ 7s, 30 cycles |
| **#5** | **Ee2F - ER** | 1398bp | annealing: 54.5^o^C, 10s extension: 70^o^C/ 26s, 45 cycles |

**Table S2.** Primers and conditions used for the amplification of *cbh7* gene through PCR (#1), removal of introns (#2-3) and final overlapping PCR (#4). Colored sequences represent the restriction sites of *ClaI* and *XbaI* enzymes (red, purple), as well as the complementary DNA fragments that allowed the hybridization reaction and the amplification of the generated “*fusion*” fragment by overlapping PCR (*blue: annealing, green: overhang*).

**(A)**

|  | primer sequence | |
| --- | --- | --- |
| *Tt*CBH7_109566**F** (30 bp) | 5' **GCA TCG ATG CAG AAC GCC TGC ACT CTG ACC** 3' | |
|  | *ClaI* | |
| *Tt*CBH7_109566**R** (32 bp) | 5' **CGT CTA GAA GGC ACT GCG AGT ACC AGT CAT TC** 3' | |
|  | *XbaI* | |
|  | | primer sequence (OE-PCR) |
| *Tt*CBH7_109566**e2F**  (21 bp) | | 5' **TGT TCC AGC TCC TCG GCA ACG** 3' |
| *Tt*CBH_109566**e1R** (38 bp) | | 5' **CGA GGA GCT GGA ACA TCT GGT ACT TGG TGT CGC TCT CC** 3' |

**(B)**

| **PCR** | **Primers** | **Fragment targeted** | **Conditions** |
| --- | --- | --- | --- |
| **#1** | **EF - ER** | 2001 bp | annealing: 60 ^o^C /10s extension: 70 ^o^C /33s, 30 cycles |
| **#2** | **EF - Ee1R** | 409 bp | annealing: 60 ^o^C /10s extension: 70 ^o^C /4s, 30 cycles |
| **#3** | **Ee2F - ER** | 1172 bp | annealing: 60 ^o^C C/10s extension: 56 ^o^C /12s, 30 cycles |
| **#4** | **EF - ER** | 1581 bp | annealing: 59 ^o^C /10s extension: 59 ^o^C /25s, 45 cycles |

**Table S3.** Properties of *Tt*CBH6 and *Tt*CBH7 obtained from genome analysis.

| **Genome Portal ID** | 66729 | 109566 |
| --- | --- | --- |
| **Chromosome** | 2: 46305-48489 | 1: 9753507-9755507 |
| **Family** | Glycoside hydrolase 6 | Glycoside hydrolase 7 |
| **Domains** | CBM_1, [Pfam: PR00734, InterProScan] | CBM_1, [Pfam: PR00734, InterProScan] |
| **Gene (translation)** | 1449 bp | 1581bp |
| **Gene (trancription) [3’UTP, 5’UTP]** | 1832 bp | 1934 bp |
| **Protein** | 465 aa | 526 aa |
| **Exons** | 4 | 2 |
| **Secretion signal** | MAKKLFITAALAAAVLA (17 aa) | MYAKFATLAALVAGAAA (17 aa) |
| **Theoretical predicted MW** | 49.41 kDa | 56 kDa |
| **theoretical pI** | 5.28 | 4.95 |
| **Glucosylation sites N-Glyc** | 1 | 1 |
| **Glucosylation sites O-Glyc** | 44 | 23 |

**Table S4.** Yields from *birch* hydrolysis at 24 and 48 h ( *#*1 experimental design).

|  |  |  |  |  | **24h** | **48h** | **24h** | **48h** |
| --- | --- | --- | --- | --- | --- | --- | --- | --- |
| Run | A:EG5 | B:EG7 | C:CBH6 | D:CBH7 | *cellobiose* | *cellobiose* | *% hydrolysis* | *% hydrolysis* |
|  |  |  |  |  | *mg/mL* | *mg/mL* |  |  |
| **1** | 0.25 | 0.2 | 0.05 | 0.5 | 2.92 | 5.65 | 19.34 | 37.43 |
| **2** | 0.1 | 0.05 | 0.05 | 0.8 | 3.35 | 5.99 | 22.17 | 39.68 |
| **3** | 0.1 | 0.1 | 0.3 | 0.5 | 3.63 | 5.25 | 24.04 | 34.77 |
| **4** | 0.221 | 0.05 | 0.193 | 0.537 | 3.62 | 4.46 | 23.98 | 29.56 |
| **5** | 0.3 | 0.055 | 0.145 | 0.5 | 3.95 | 4.61 | 26.15 | 30.50 |
| **6** | 0.182 | 0.122 | 0.135 | 0.56 | 2.92 | 4.88 | 19.37 | 32.31 |
| **7** | 0.1 | 0.2 | 0.2 | 0.5 | 2.63 | 3.74 | 17.45 | 24.75 |
| **8** | 0.1 | 0.2 | 0.05 | 0.65 | 2.60 | 4.91 | 17.23 | 32.53 |
| **9** | 0.1 | 0.05 | 0.211 | 0.639 | 3.25 | 3.94 | 21.51 | 26.11 |
| **10** | 0.156 | 0.102 | 0.05 | 0.693 | 3.16 | 5.66 | 20.90 | 37.51 |
| **11** | 0.175 | 0.05 | 0.275 | 0.5 | 3.51 | 2.82 | 23.23 | 18.66 |
| **12** | 0.179 | 0.193 | 0.05 | 0.578 | 2.91 | 5.18 | 19.26 | 34.31 |
| **13** | 0.27 | 0.05 | 0.05 | 0.63 | 3.72 | 5.78 | 24.67 | 38.29 |
| **14** | 0.176 | 0.051 | 0.133 | 0.64 | 3.54 | 4.47 | 23.45 | 29.61 |
| **15** | 0.27 | 0.05 | 0.05 | 0.63 | 3.87 | 5.06 | 25.63 | 33.52 |
| **16** | 0.25 | 0.2 | 0.05 | 0.5 | 2.96 | 4.72 | 19.60 | 31.29 |
| **17** | 0.1 | 0.05 | 0.132 | 0.718 | 3.33 | 4.39 | 22.08 | 29.08 |
| **18** | 0.1 | 0.2 | 0.2 | 0.5 | 2.79 | 3.37 | 18.47 | 22.33 |
| **19** | 0.3 | 0.055 | 0.145 | 0.5 | 3.91 | 4.53 | 25.89 | 30.02 |
| **20** | 0.1 | 0.05 | 0.05 | 0.8 | 3.53 | 5.20 | 23.37 | 34.42 |

**Table S5.** Yields from *spruce* hydrolysis at 24 and 48 h ( #1 experimental design).

|  |  |  |  |  | **24h** | **48h** | **24h** | **48h** |
| --- | --- | --- | --- | --- | --- | --- | --- | --- |
| Run | A:EG5 | B:EG7 | C:CBH6 | D:CBH7 | *cellobiose* | *cellobiose* | *% hydrolysis* | *% hydrolysis* |
|  |  |  |  |  | *mg/mL* | *mg/mL* |  |  |
| **1** | 0.25 | 0.2 | 0.05 | 0.5 | 3.01 | 5.16 | 20.27 | 34.72 |
| **2** | 0.1 | 0.05 | 0.05 | 0.8 | 2.84 | 5.24 | 19.12 | 35.31 |
| **3** | 0.1 | 0.1 | 0.3 | 0.5 | 2.60 | 4.47 | 17.51 | 30.08 |
| **4** | 0.221 | 0.05 | 0.193 | 0.537 | 2.90 | 4.96 | 19.52 | 33.42 |
| **5** | 0.3 | 0.055 | 0.145 | 0.5 | 2.97 | 5.41 | 19.98 | 36.41 |
| **6** | 0.182 | 0.122 | 0.135 | 0.56 | 3.09 | 5.72 | 20.82 | 38.48 |
| **7** | 0.1 | 0.2 | 0.2 | 0.5 | 2.69 | 4.65 | 18.12 | 31.32 |
| **8** | 0.1 | 0.2 | 0.05 | 0.65 | 2.88 | 5.08 | 19.38 | 34.22 |
| **9** | 0.1 | 0.05 | 0.211 | 0.639 | 2.75 | 4.78 | 18.52 | 32.22 |
| **10** | 0.156 | 0.102 | 0.05 | 0.693 | 3.05 | 5.42 | 20.55 | 36.49 |
| **11** | 0.175 | 0.05 | 0.275 | 0.5 | 2.78 | 4.92 | 18.69 | 33.13 |
| **12** | 0.179 | 0.193 | 0.05 | 0.578 | 2.93 | 5.04 | 19.76 | 33.91 |
| **13** | 0.27 | 0.05 | 0.05 | 0.63 | 3.36 | 5.39 | 22.65 | 36.27 |
| **14** | 0.176 | 0.051 | 0.133 | 0.64 | 3.37 | 5.37 | 22.66 | 36.16 |
| **15** | 0.27 | 0.05 | 0.05 | 0.63 | 3.31 | 5.28 | 22.30 | 35.54 |
| **16** | 0.25 | 0.2 | 0.05 | 0.5 | 3.06 | 4.91 | 20.61 | 33.05 |
| **17** | 0,1 | 0.05 | 0.132 | 0.718 | 2.98 | 5.27 | 20.04 | 35.47 |
| **18** | 0,1 | 0.2 | 0.2 | 0.5 | 2.74 | 4.57 | 18.43 | 30.80 |
| **19** | 0,3 | 0.055 | 0.145 | 0.5 | 2.88 | 5.38 | 19.37 | 36.23 |
| **20** | 0,1 | 0.05 | 0.05 | 0.8 | 3.03 | 5.42 | 20.41 | 36.49 |

**Table S6.** Yields from *birch* and *spruce* hydrolysis at 48 h ( #2 experimental design).

|  |  |  |  |  | **birch** | **spruce** | **birch** | **spruce** |
| --- | --- | --- | --- | --- | --- | --- | --- | --- |
| Run | A:EG5 | B:EG7 | C:EG9 | D:EG6 | *cellobiose* | *cellobiose* | *% hydrolysis* | *% hydrolysis* |
|  |  |  |  |  | *mg/mL* | *mg/mL* |  |  |
| **1** | 0.38 | 0.55 | 0.04 | 0.03 | 5.25 | 5.21 | 34.74 | 35.07 |
| **2** | 0.20 | 0.70 | 0.06 | 0.05 | 6.14 | 5.10 | 40.65 | 34.34 |
| **3** | 0.35 | 0.50 | 0.10 | 0.06 | 4.90 | 5.04 | 32.42 | 33.94 |
| **4** | 0.20 | 0.69 | 0.10 | 0.01 | 6.28 | 5.16 | 41.60 | 34.76 |
| **5** | 0.27 | 0.70 | 0.02 | 0.01 | 5.91 | 5.48 | 39.15 | 36.87 |
| **6** | 0.32 | 0.55 | 0.06 | 0.07 | 5.47 | 4.16 | 36.25 | 27.99 |
| **7** | 0.25 | 0.63 | 0.06 | 0.05 | 5.24 | 3.74 | 34.71 | 25.21 |
| **8** | 0.45 | 0.50 | 0.02 | 0.03 | 5.78 | 3.90 | 38.29 | 26.26 |
| **9** | 0.33 | 0.60 | 0.06 | 0.01 | 5.60 | 3.64 | 37.06 | 24.50 |
| **10** | 0.45 | 0.50 | 0.02 | 0.03 | 5.78 | 4.37 | 38.29 | 29.45 |
| **11** | 0.20 | 0.68 | 0.02 | 0.10 | 5.44 | 5.50 | 36.03 | 37.01 |
| **12** | 0.20 | 0.60 | 0.10 | 0.10 | 5.53 | 4.79 | 36.60 | 32.24 |
| **13** | 0.20 | 0.60 | 0.10 | 0.10 | 5.53 | 4.64 | 36.60 | 31.26 |
| **14** | 0.20 | 0.68 | 0.02 | 0.10 | 5.97 | 4.80 | 39.56 | 32.32 |
| **15** | 0.30 | 0.50 | 0.10 | 0.10 | 5.66 | 4.03 | 37.47 | 27.11 |
| **16** | 0.38 | 0.50 | 0.02 | 0.10 | 5.92 | 4.02 | 39.22 | 27.07 |
| **17** | 0.27 | 0.57 | 0.06 | 0.10 | 5.73 | 4.55 | 37.97 | 30.62 |
| **18** | 0.27 | 0.70 | 0.02 | 0.01 | 5.93 | 5.28 | 39.26 | 35.55 |
| **19** | 0.33 | 0.60 | 0.02 | 0.06 | 5.60 | 3.61 | 37.06 | 24.28 |
| **20** | 0.20 | 0.69 | 0.10 | 0.01 | 6.28 | 5.45 | 41.60 | 36.69 |

**Table S7.** Final equations of actual components of #1 experimental design

| **Birch** |  |  |  |  |  |  |  | |
| --- | --- | --- | --- | --- | --- | --- | --- | --- |
| 24h: Quadratic model  R^2^ = 0.9484, p-value < 0.0001 | | | | 48h: Quadratic model  R^2^ = 0.7932, p-value 0.0168 | | |  |  |
| cellobiose = | |  |  | cellobiose = | |  |  | |
| +16.80039 | * EG5 |  |  | +30.77433 | * EG5 |  |  | |
| -1.15682 | * EG7 |  |  | -135.37455 | * EG7 |  |  | |
| +14.39950 | * CBH6 | |  | +21.28917 | * CBH6 | |  | |
| +4.15399 | * CBH7 | |  | +5.90992 | * CBH7 | |  | |
| -16.63579 | * EG5 * EG7 | |  | +160.27257 | * EG5 * EG7 | |  | |
| -29.34325 | * EG5 * CBH6 | |  | -82.80772 | * EG5 * CBH6 | |  | |
| -16.57553 | * EG5 * CBH7 | |  | -40.55917 | * EG5 * CBH7 | |  | |
| -7.39255 | * EG7 * CBH6 | |  | +156.35049 | * EG7 * CBH6 | |  | |
| -0.72019 | * EG7 * CBH7 | |  | +178.82617 | * EG7 * CBH7 | |  | |
| -15.56990 | * CBH6 * CBH7 | |  | -32.12065 | * CBH6 * CBH7 | |  | |
|  |  |  |  |  |  |  |  | |
| **Spruce** |  |  |  |  |  |  |  | |
| 24h: Quadratic model  R^2^ = 0.8655, p-value 0.0025 | | | | 48h: Quadratic model  R^2^ = 0.8796, p-value 0.0015 | | | |  |
| cellobiose = | |  |  | cellobiose = | |  |  |  |
| -9.24485 | * EG5 |  |  | +0.53371 | * EG5 |  |  |  |
| +4.67174 | * EG7 |  |  | -45.24308 | * EG7 |  |  |  |
| -2.91388 | * CBH6 | |  | -10.29994 | * CBH6 | |  |  |
| +1.96287 | * CBH7 | |  | +4.78266 | * CBH7 | |  |  |
| +10.55394 | * EG5 * EG7 | |  | +70.19550 | * EG5 * EG7 | |  |  |
| +14.48936 | * EG5 * CBH6 | |  | +34.70917 | * EG5 * CBH6 | |  |  |
| +23.63365 | * EG5 * CBH7 | |  | +5.00192 | * EG5 * CBH7 | |  |  |
| +4.75547 | * EG7 * CBH6 | |  | +79.17612 | * EG7 * CBH6 | |  |  |
| -3.65436 | * EG7 * CBH7 | |  | +63.46860 | * EG7 * CBH7 | |  |  |
| +8.16948 | * CBH6 * CBH7 | |  | +14.28289 | * CBH6 * CBH7 | |  |  |

**Table S8.** Final equations of actual components of #2 experimental design.

| **Birch** |  |  |  | **Spruce** |  |  |  |
| --- | --- | --- | --- | --- | --- | --- | --- |
| 48h: Quadratic model  R^2^ = 0.8189, p-value 0.0094 | | | | 48h: Special cubic model  R^2^ = 0.9409, p-value 0.0111 | | | |
| cellobiose = | |  |  | cellobiose = | = |  |  |
| +19.15538 | * EG5 |  |  | -261.80861 | * EG5 |  |  |
| +11.26938 | * CBH7 |  |  | -90.00951 | * CBH7 |  |  |
| -69.26308 | * EG9 |  |  | -1760.25389 | * EG9 |  |  |
| +128.83951 | * EG6 |  |  | -175.60650 | * EG6 |  |  |
| -36.64450 | * EG5 * CBH7 | |  | +723.70123 | * EG5 * CBH7 | |  |
| +43.39649 | * EG5 * EG9 | |  | +6012.20593 | * EG5 * EG9 | |  |
| -107.76471 | * EG5 * EG6 | |  | +2711.11224 | * EG5 * EG6 | |  |
| +85.14053 | * CBH7 * EG9 | |  | +3266.15862 | * CBH7 * EG9 | |  |
| -167.96473 | * CBH7 * EG6 | |  | +1095.75272 | * CBH7 * EG6 | |  |
| -29.80421 | * EG9 * EG6 | |  | -8891.53337 | * EG9 * EG6 | |  |
|  |  |  |  | -11068.33643 * EG5 * CBH7 * EG9 | | | |
|  |  |  |  | -7067.12105 | * EG5 * CBH7 * EG6 | |  |
|  |  |  |  | +15171.93790 * EG5 * EG9 * EG6 | | | |
|  |  |  |  | +9510.62453 | * CBH7 * EG9 * EG6 | |  |
|  |  |  |  |  |  |  |  |

**FIGURES**

**Figure S1**. Amplification of *cbh6* gene through PCR. *Intron 2* and 3 removal was done with complementary DNA primers, while *exon 1* was added as part of the **Ee2F** primer. The final OE-PCR resulted in a DNA sequence 1398 bp that is able to encode the *Tt*CBH6 protein. Primers **Ee2F** and **ER** included the *ClaI* and *XbaI* restriction sites respectively at their 5’ ends.


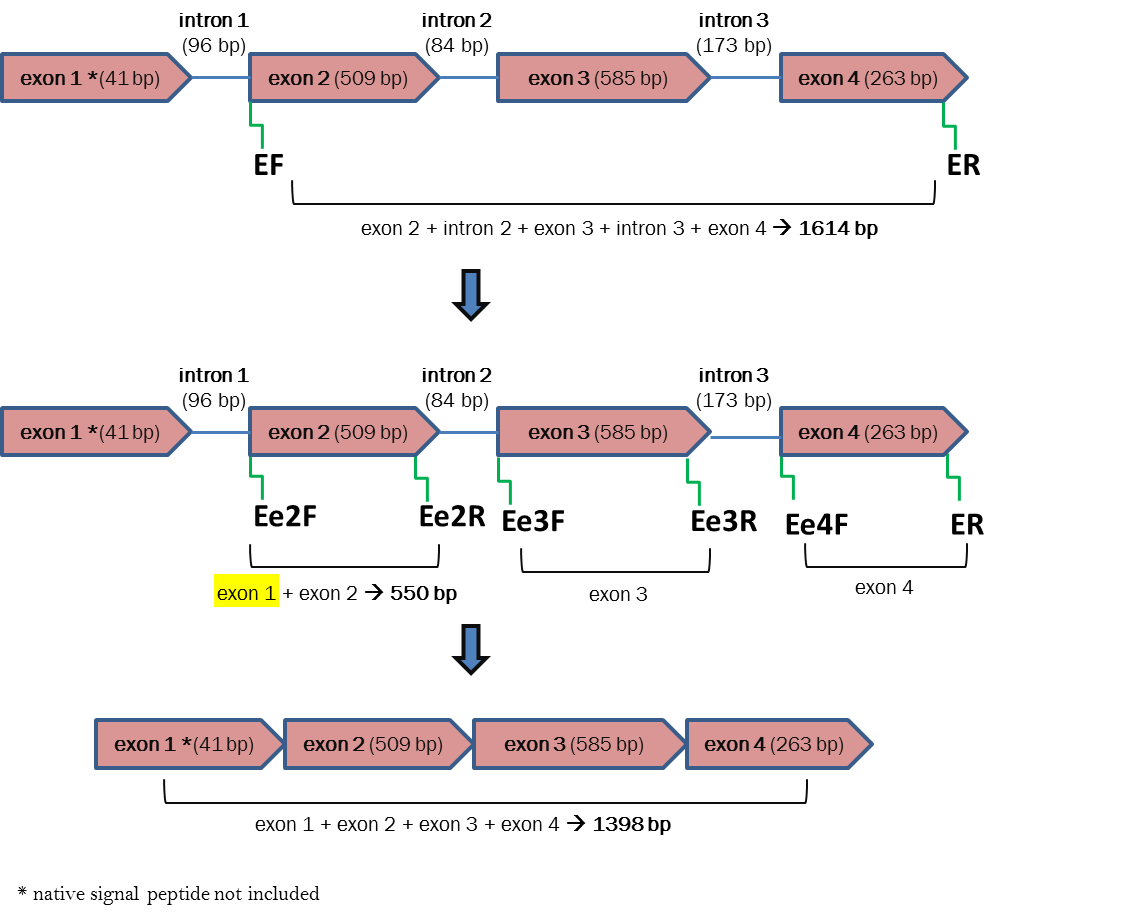


**Figure S2.** SDS-PAGE of *Tt*CBH7. *Lane 1*: Novex® sharp pre-stained protein marker, *Lane 2:* samples from culture medium, where BMMY was used as substrate, with addition of 10% w/v ammonium sulfate. Signs of proteolysis are dominant, as protein exhibit lower molecular weight, run as a “smear” and smaller size molecules appear. *Lanes 3-4:* samples of *Tt*CBH7 from culture medium with addition of 20% w/v ammonium sulfate, after ultrafitration *(3)* and after purification with IMAC *(4)*. The enzyme appears full length sized and homogenous.


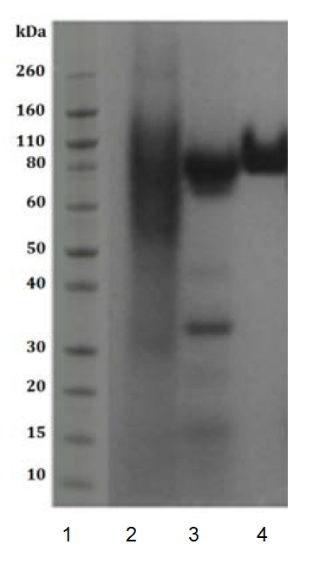


**Figure S3.** Predicted vs actual values

**(i)** Birch, 24 h **(ii)** Birch, 48 h


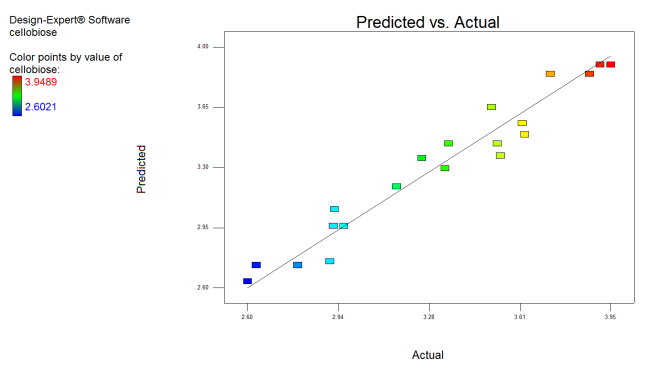

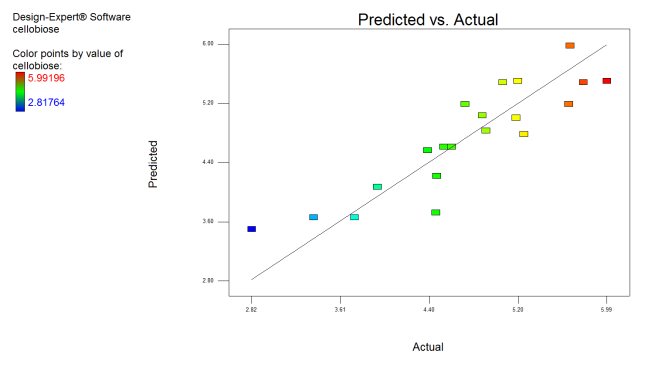


**(iii)** Spruce, 24 h **(iv)** Spruce, 48 h


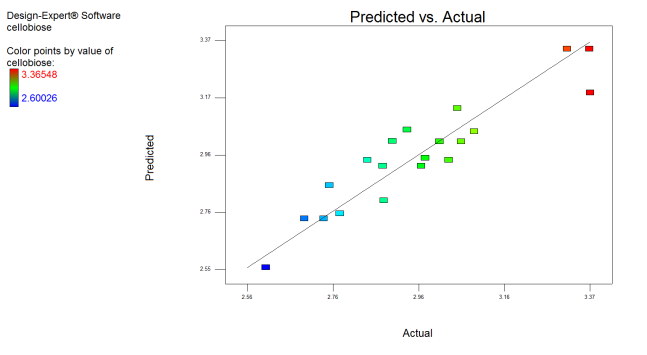

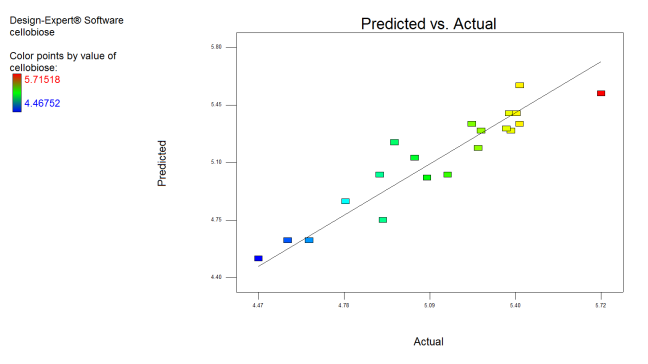


**Figure S4.** Ternary plots of **#1** experimental design showing predicted final cellobiose concentration (mg/mL) from *spruce* hydrolysis at 24 **(A)** and 48 h **(B)**, as a function of three out of four enzymes. For each plot, the forth enzyme (‘Actual Component’), has been fixed to the proportion of the point resulting in the optimal yield, as predicted by the model.

**(A) (i) (ii)**

**
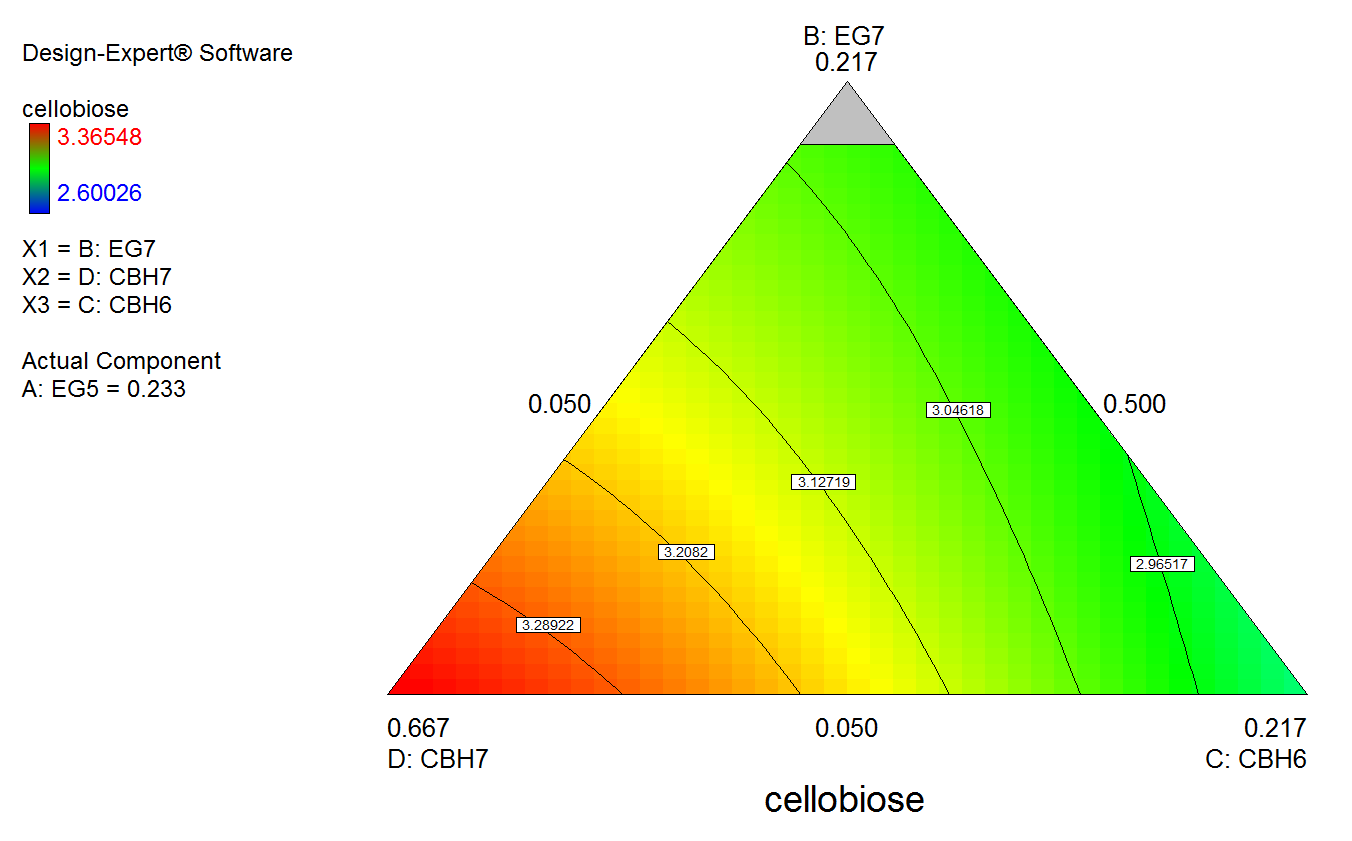

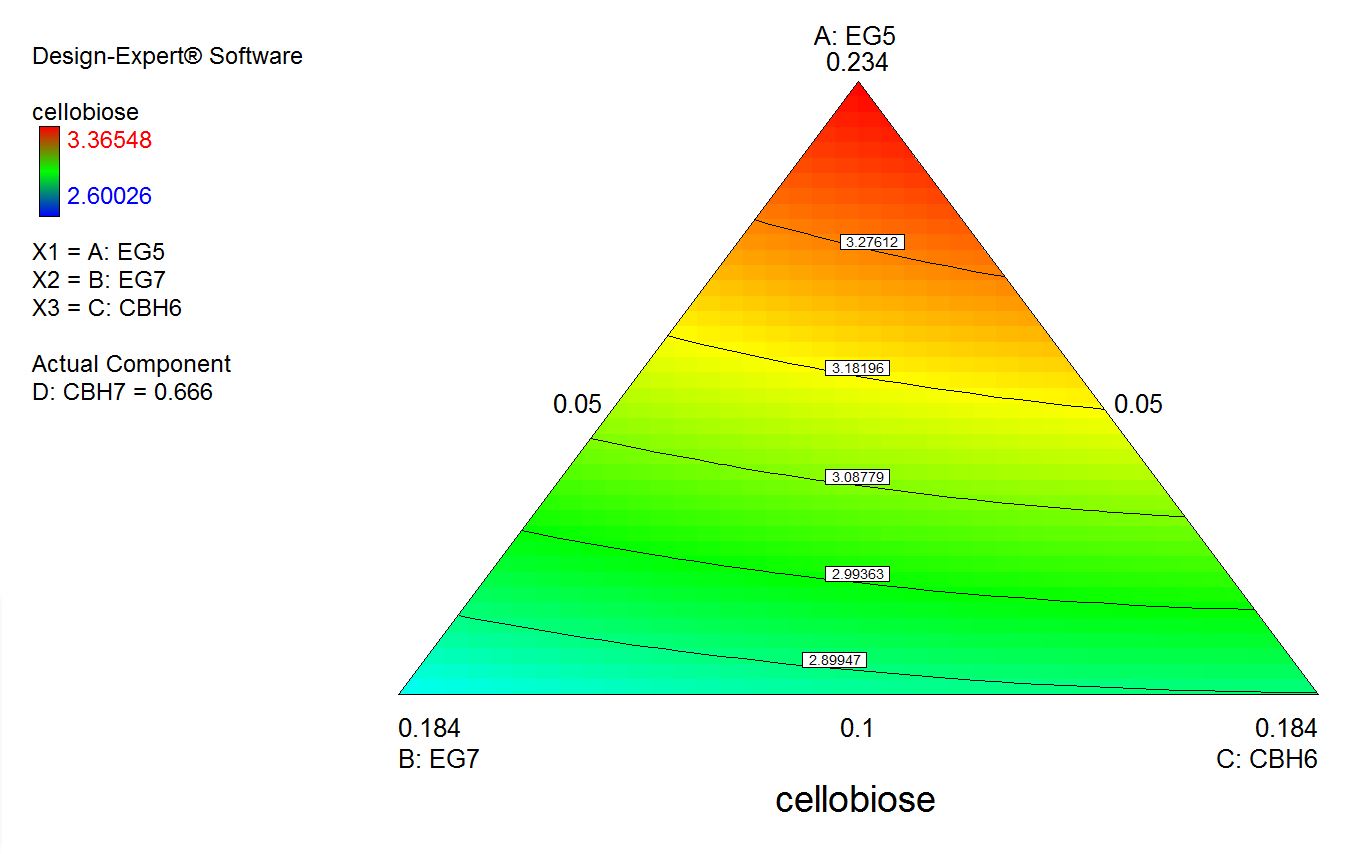
**

**(iii) (iv)**

**
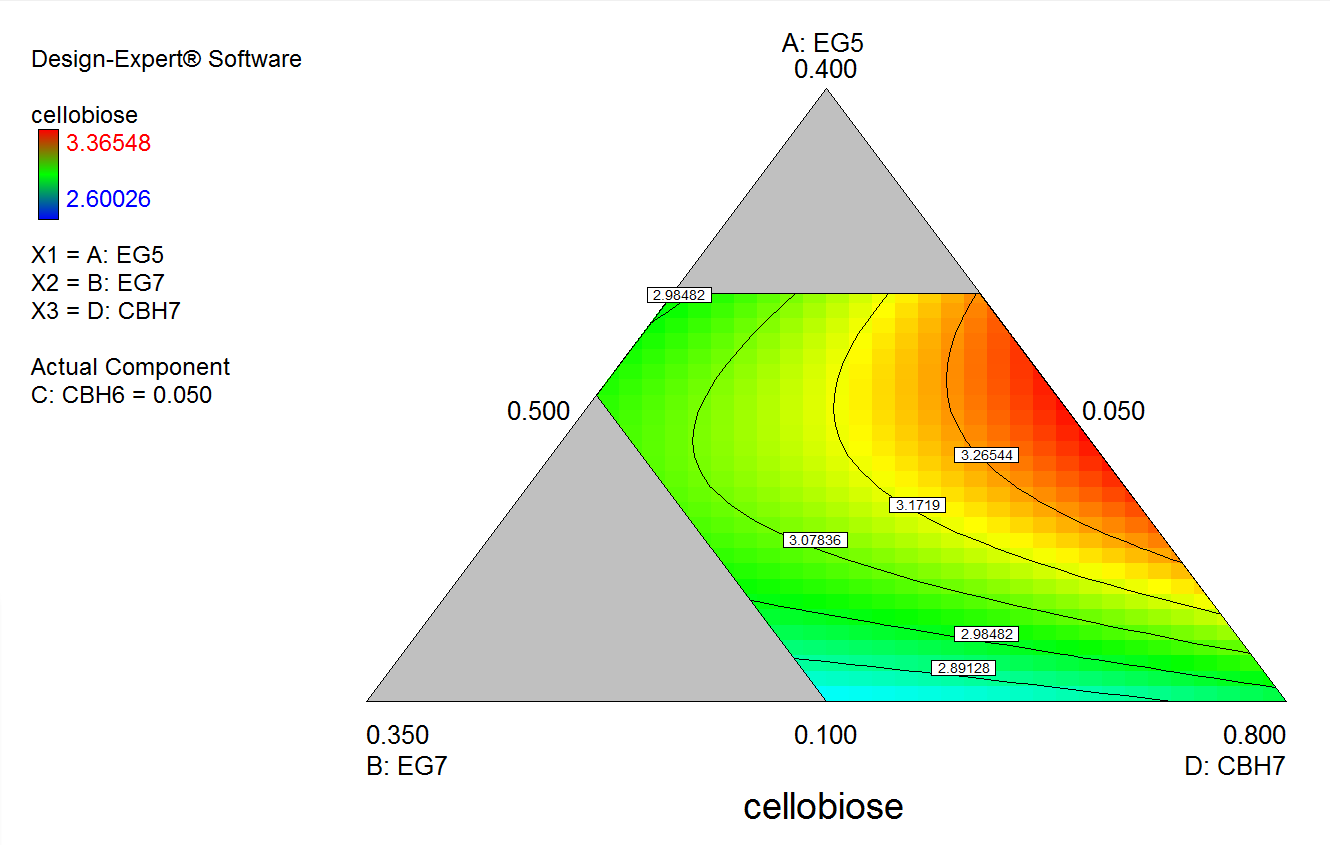

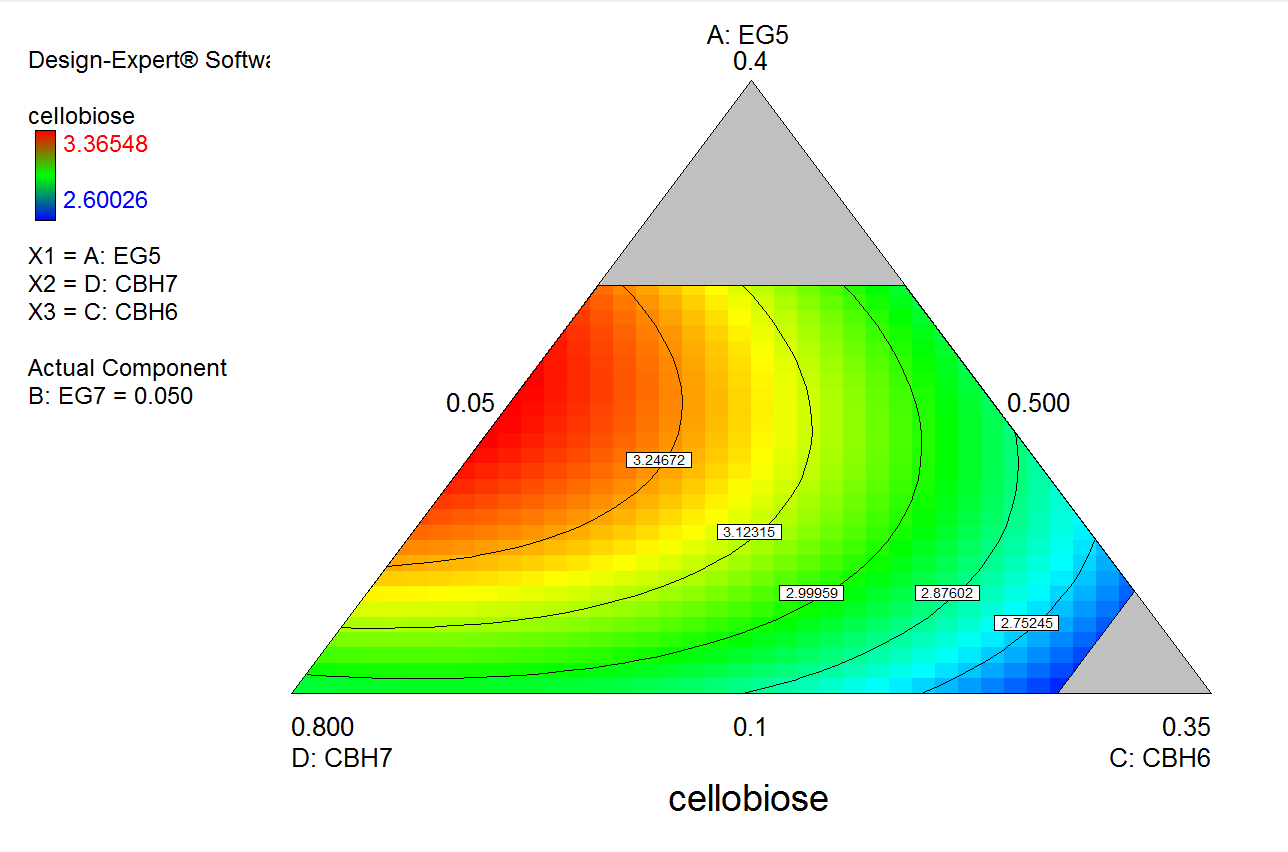
**

**(B) (i) (ii)**

**
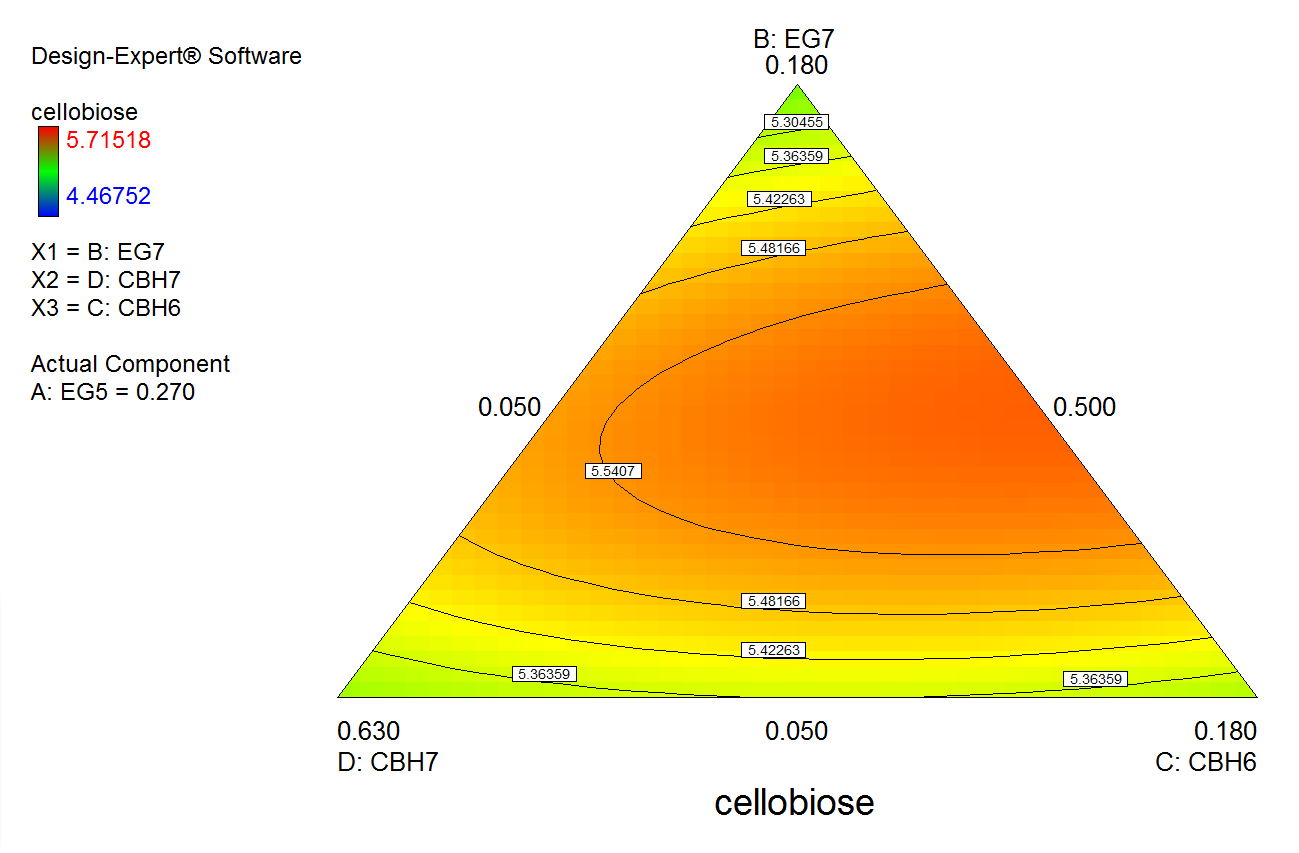

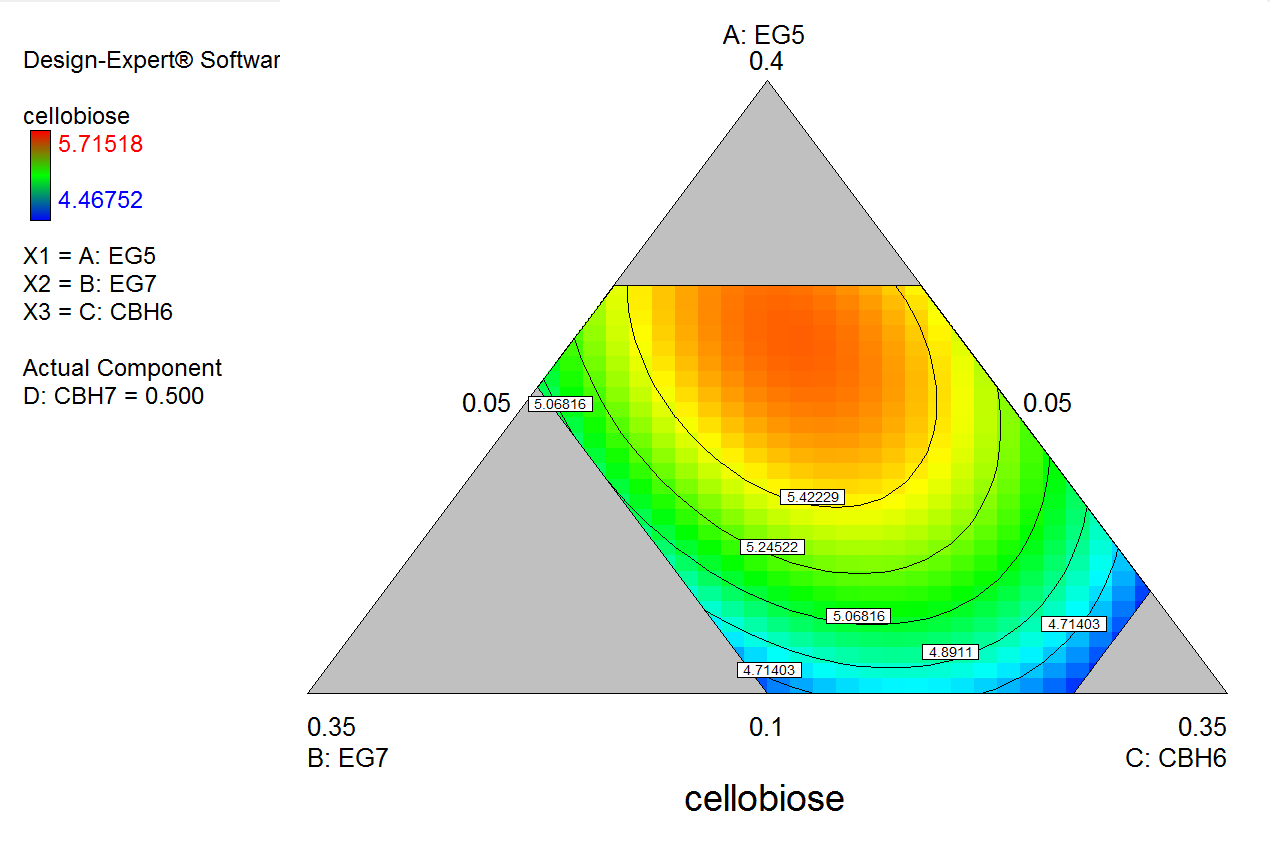
**

**(iii) (iv)**

**
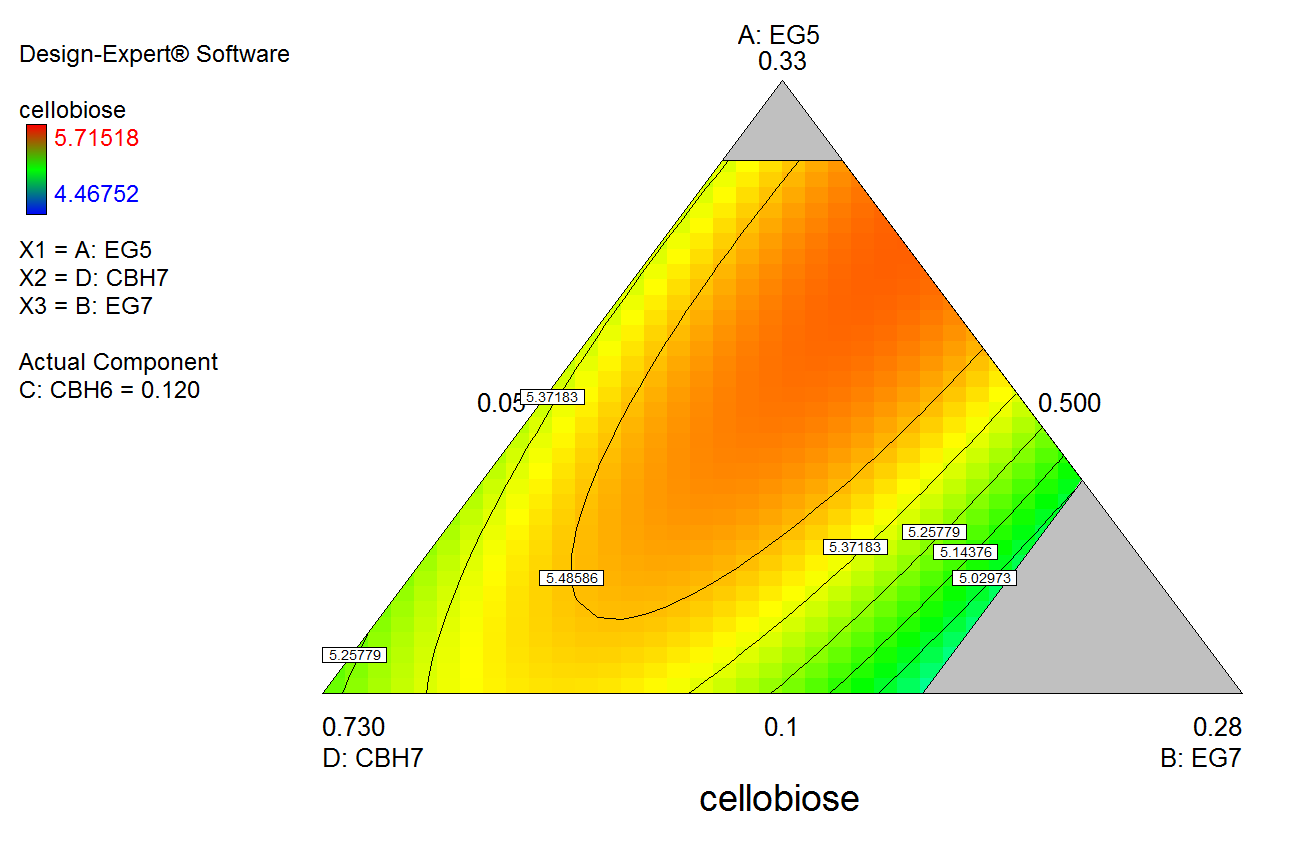

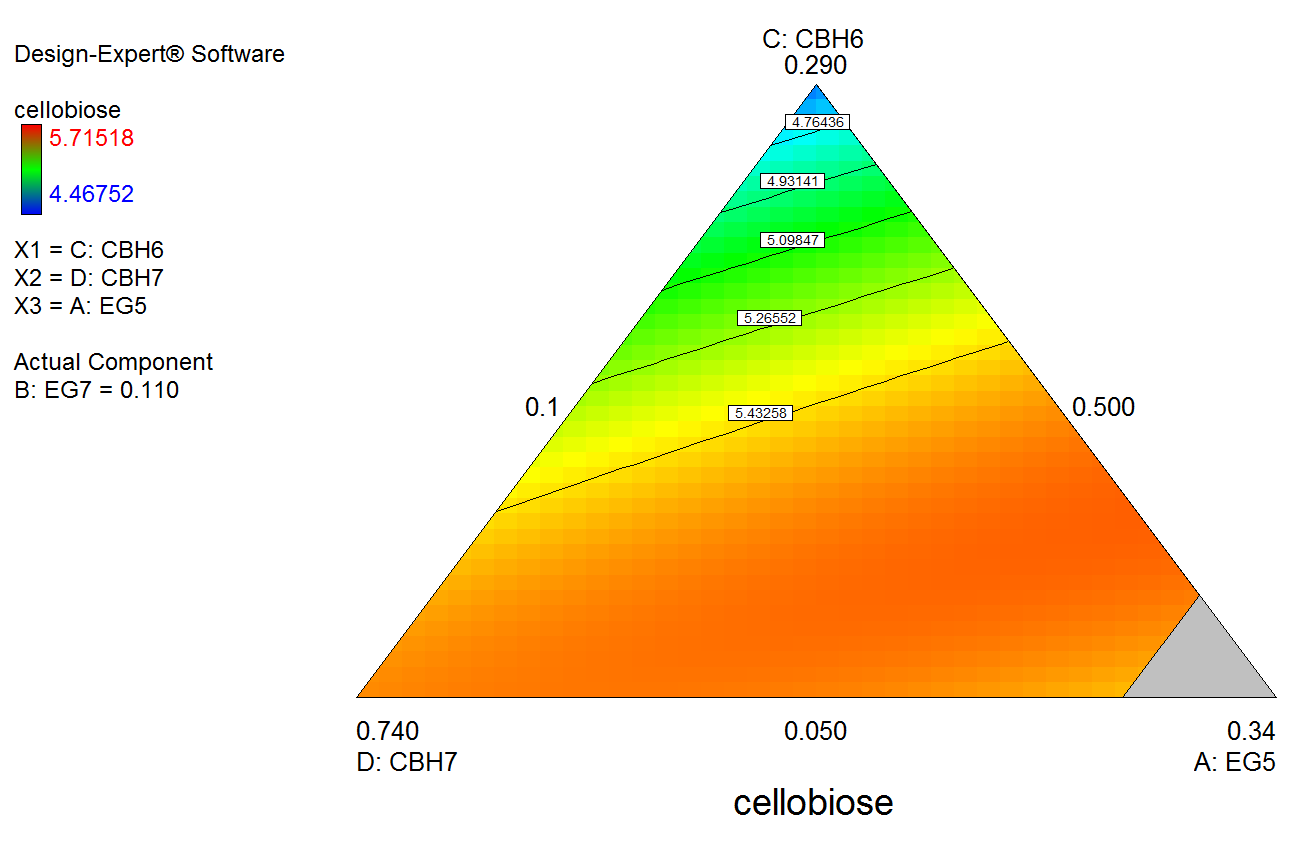
**

**Figure S5.** Ternary plots of **#1** experimental design showing predicted final cellobiose concentration (mg/mL) from *birch* hydrolysis at 24 **(A)** and 48 h **(B)**, as a function of three out of four enzymes. For each plot, the forth enzyme (‘Actual Component’), has been fixed to the proportion of the point resulting in the optimal yield, as predicted by the model.

**(A) (i) (ii)**

**
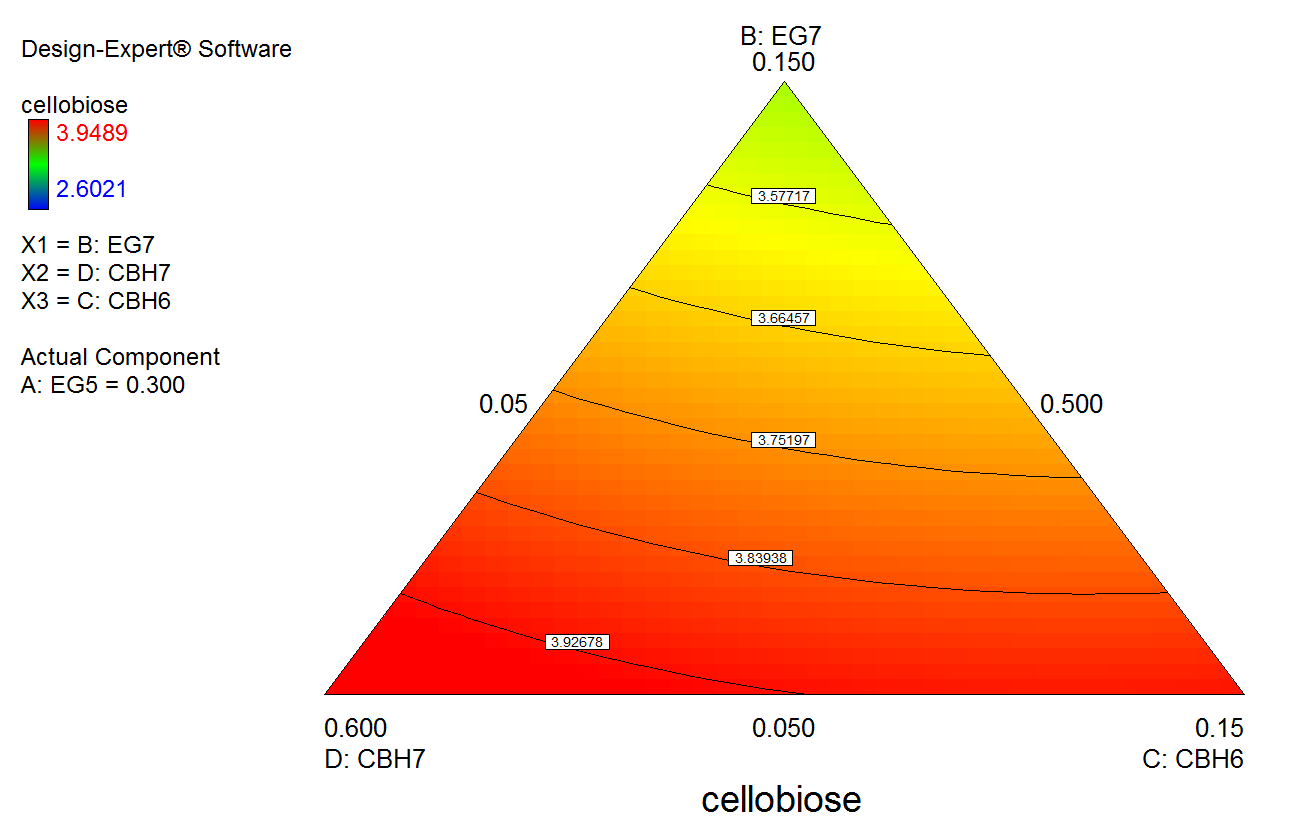

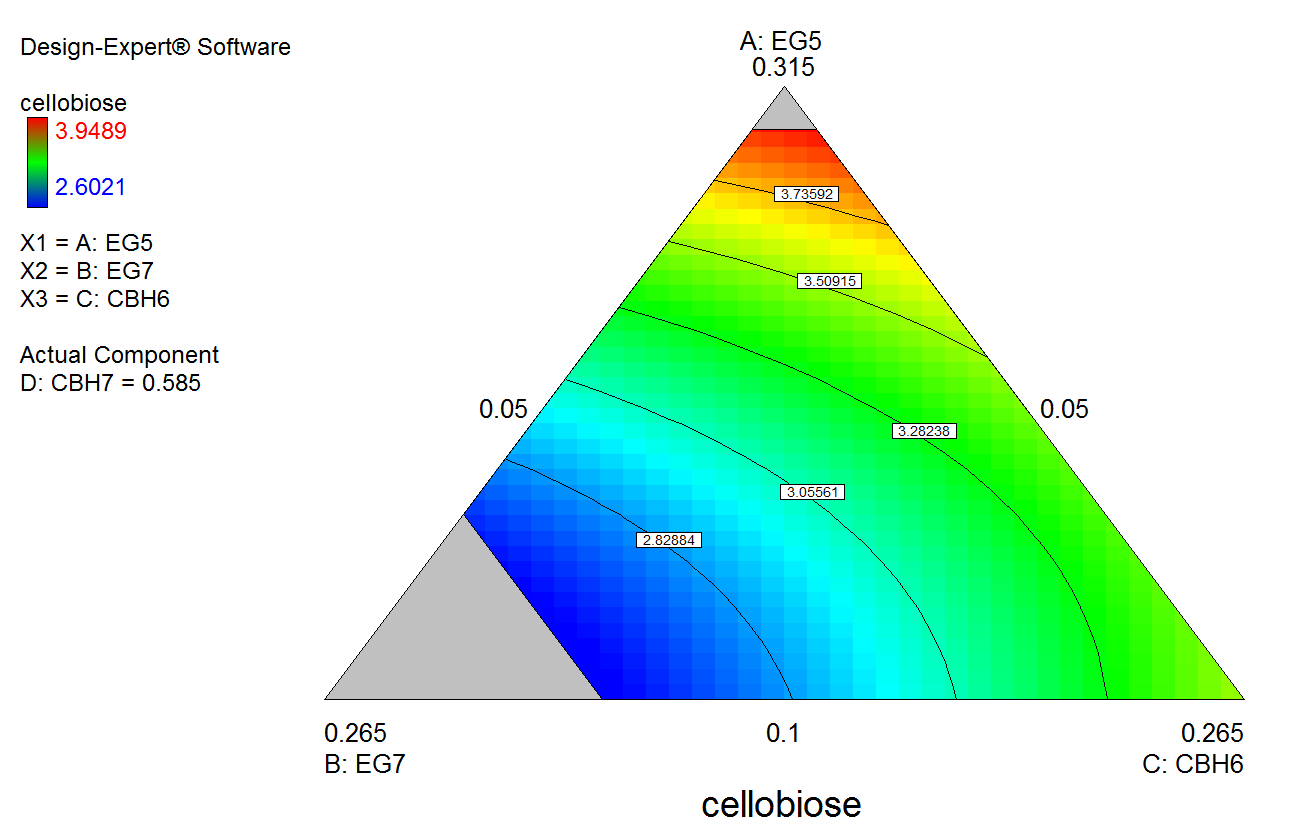
**

**(iii) (iv)**

**
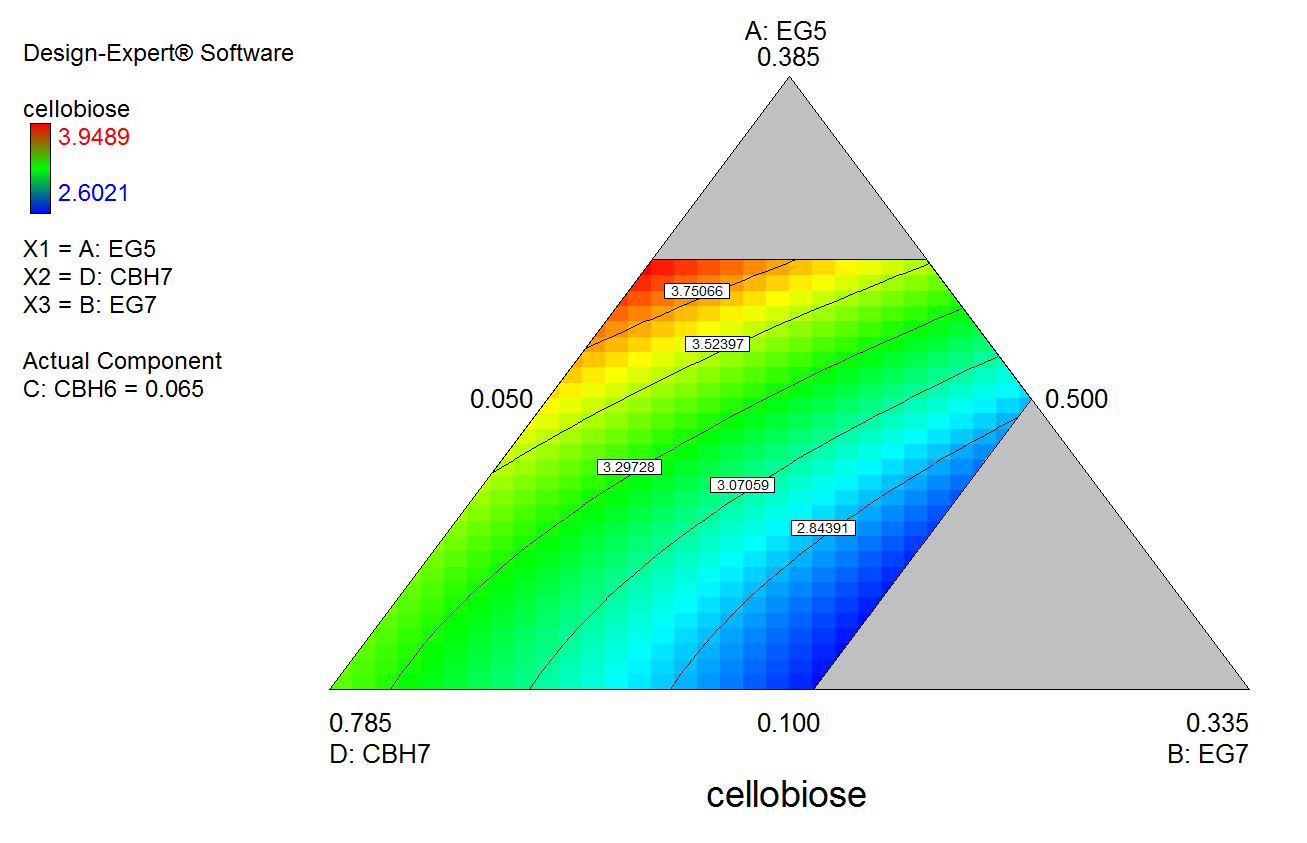

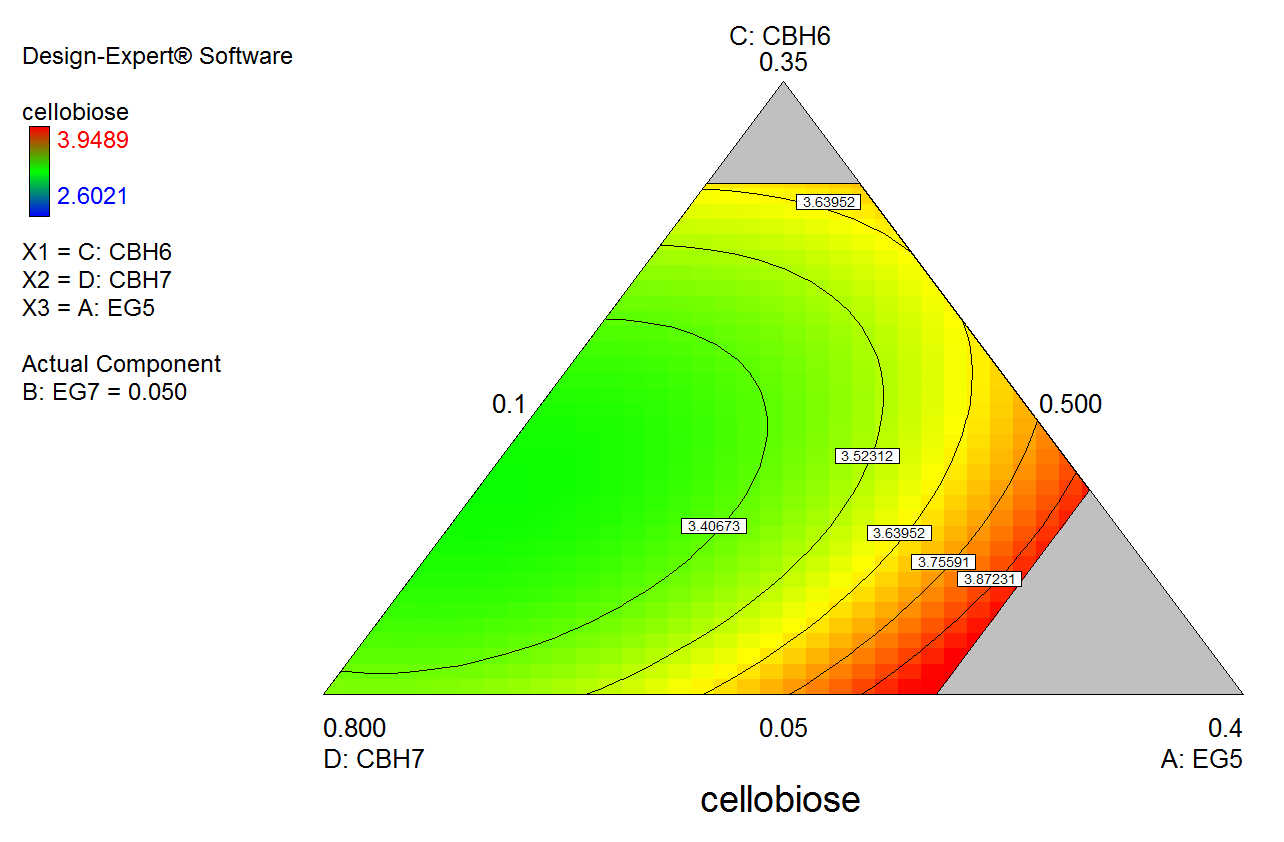
**

**(B) (i) (ii)**


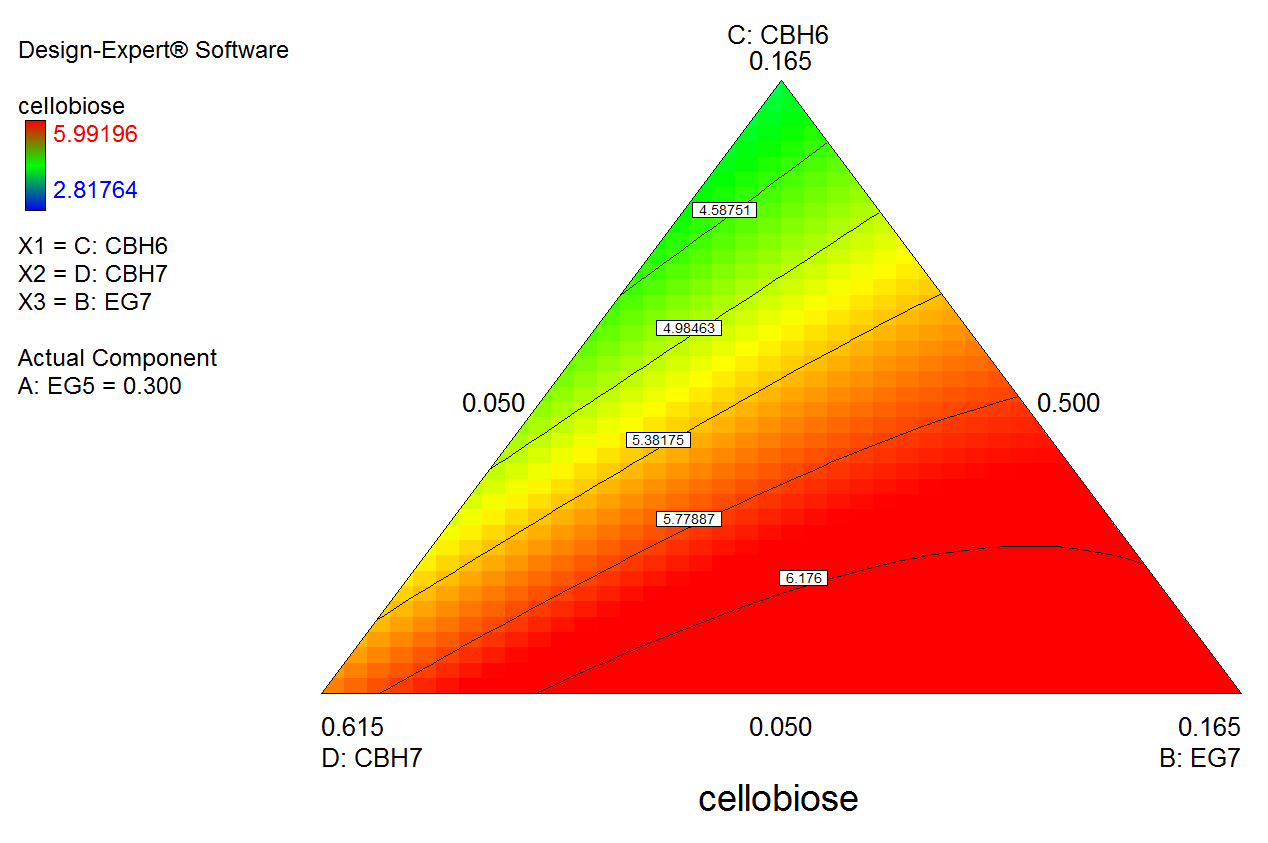

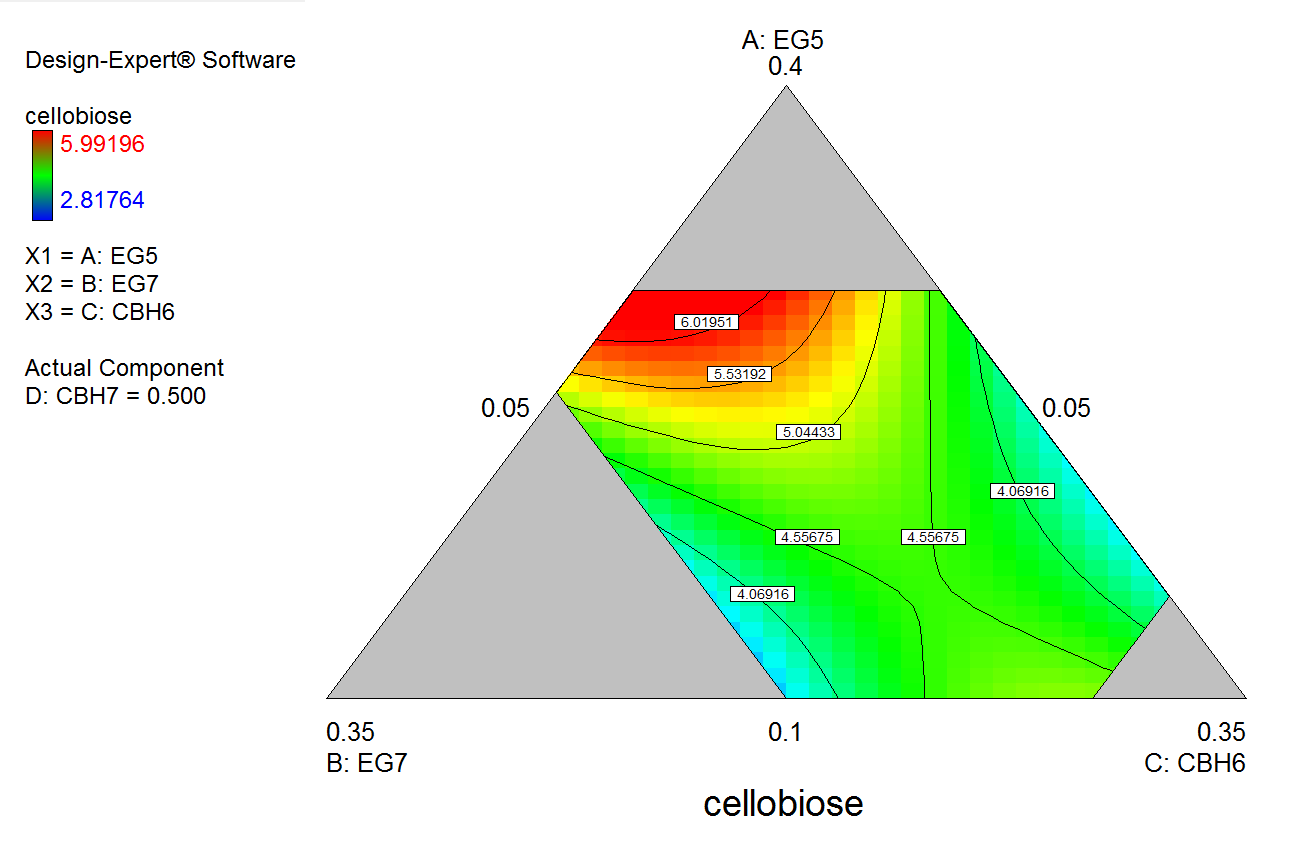


**(iii) (iv)**


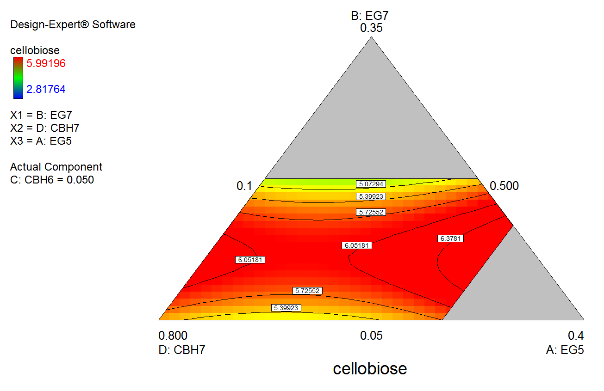

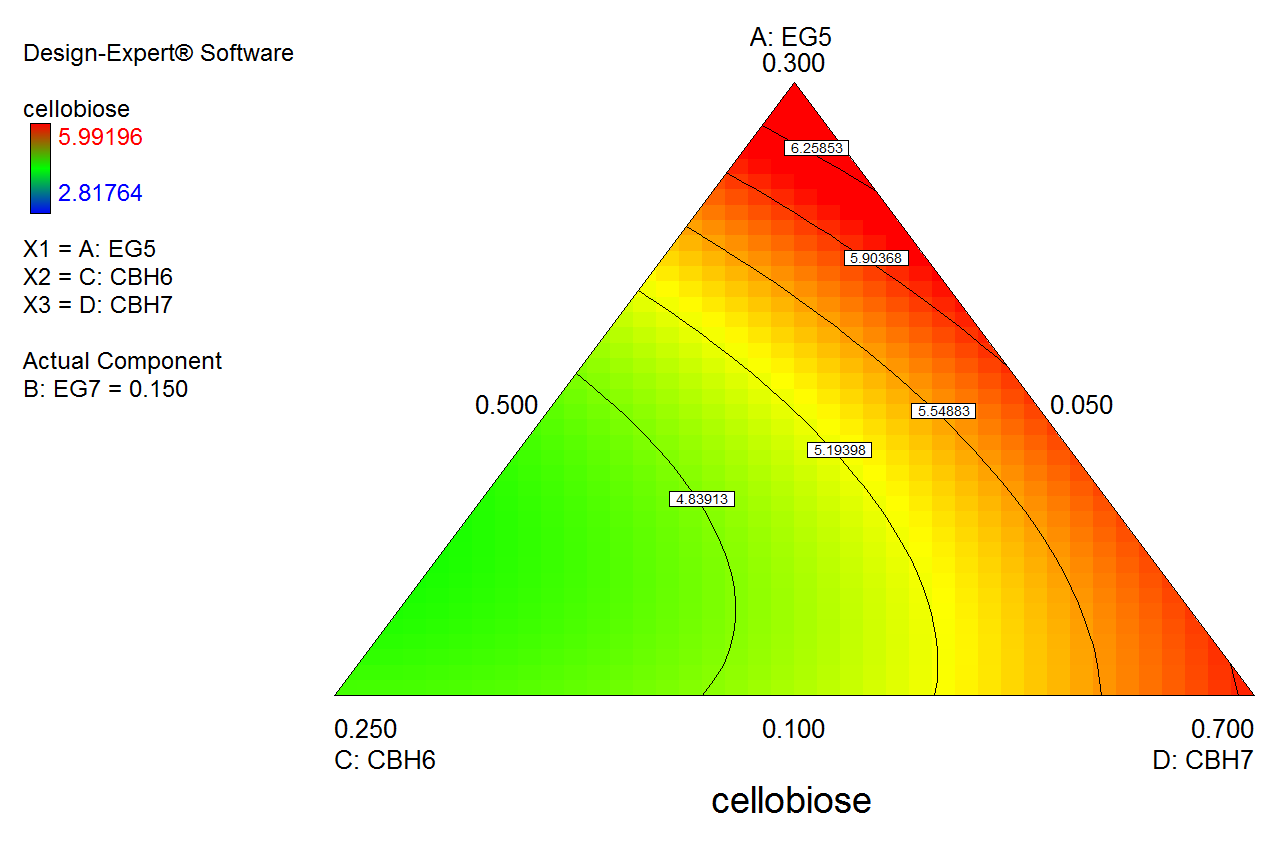


**Figure S6.** Ternary plots of **#2** experimental design showing predicted final cellobiose concentration (mg/mL) from *birch* **(A)** and *spruce* **(B)** hydrolysis at 48 h, as a function of three out of four enzymes). For each plot, the forth enzyme (‘Actual Component’) has been fixed to the proportion of the point resulting in the optimal yield, as predicted by the model.

**(A) (i) (ii)**

**
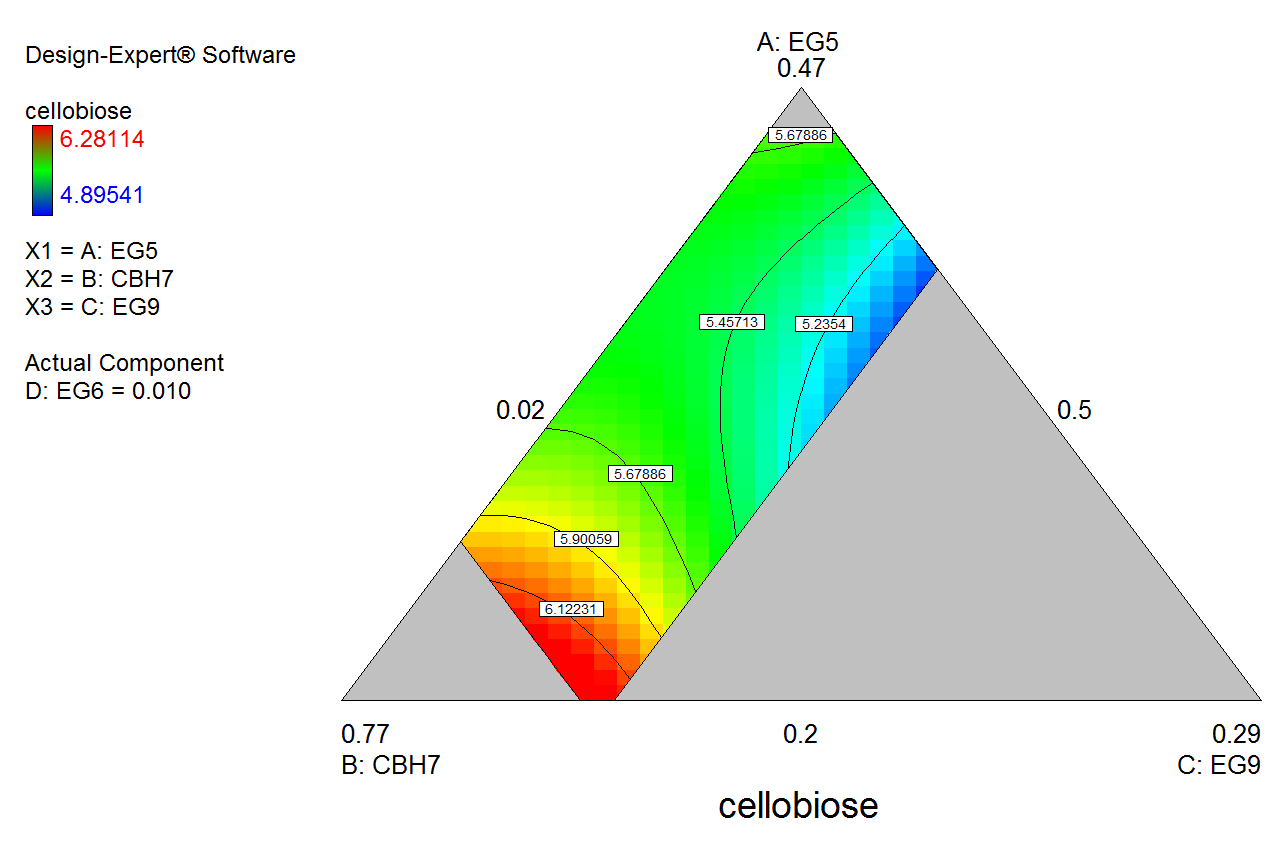

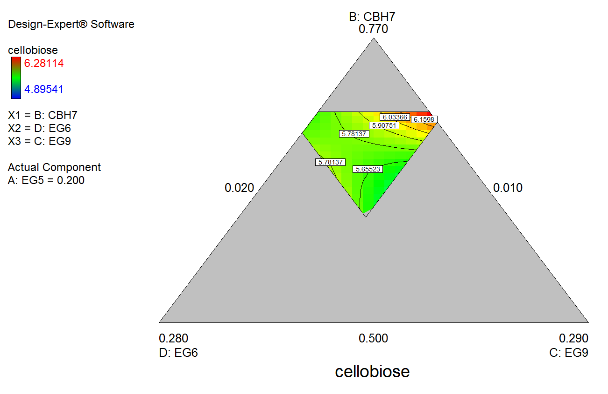
**

**(iii) (iv)**

**
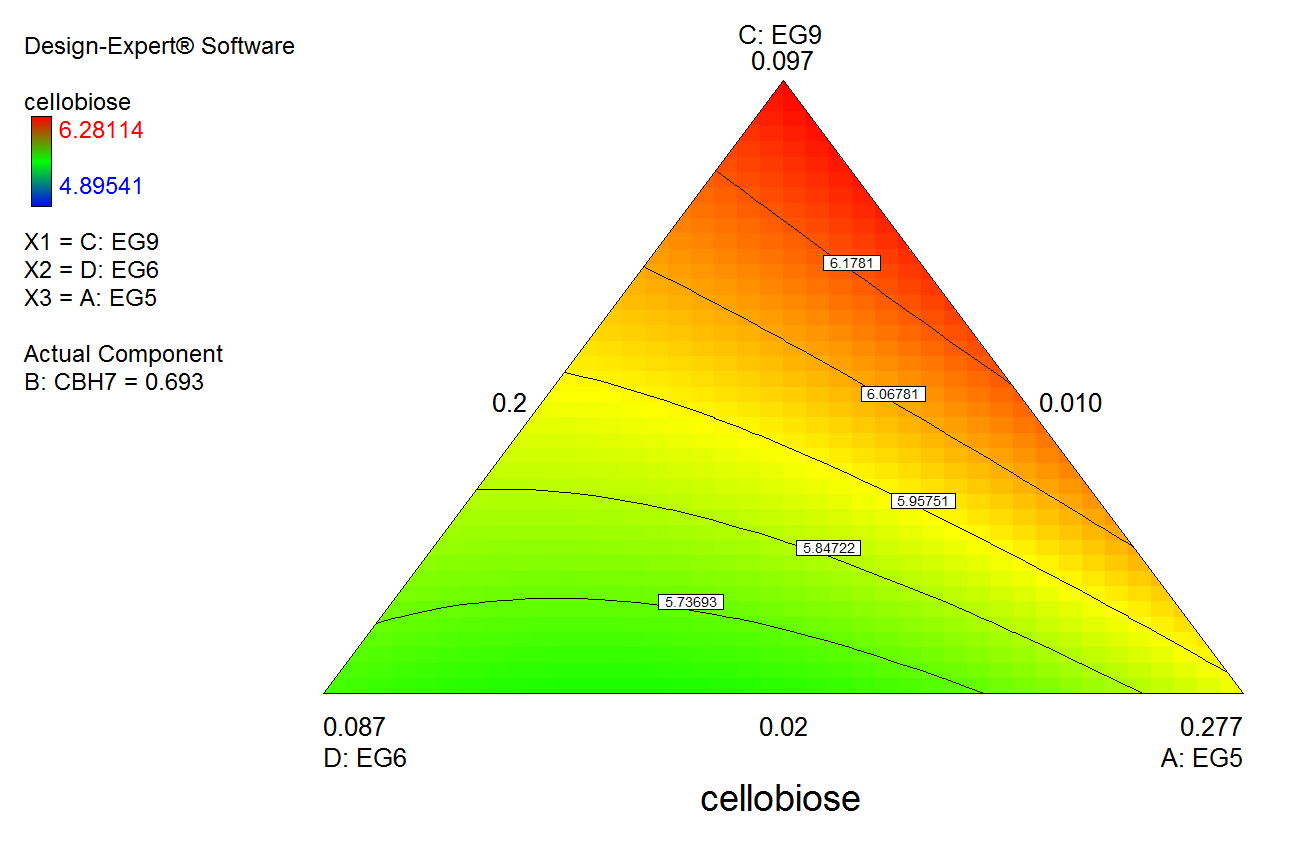

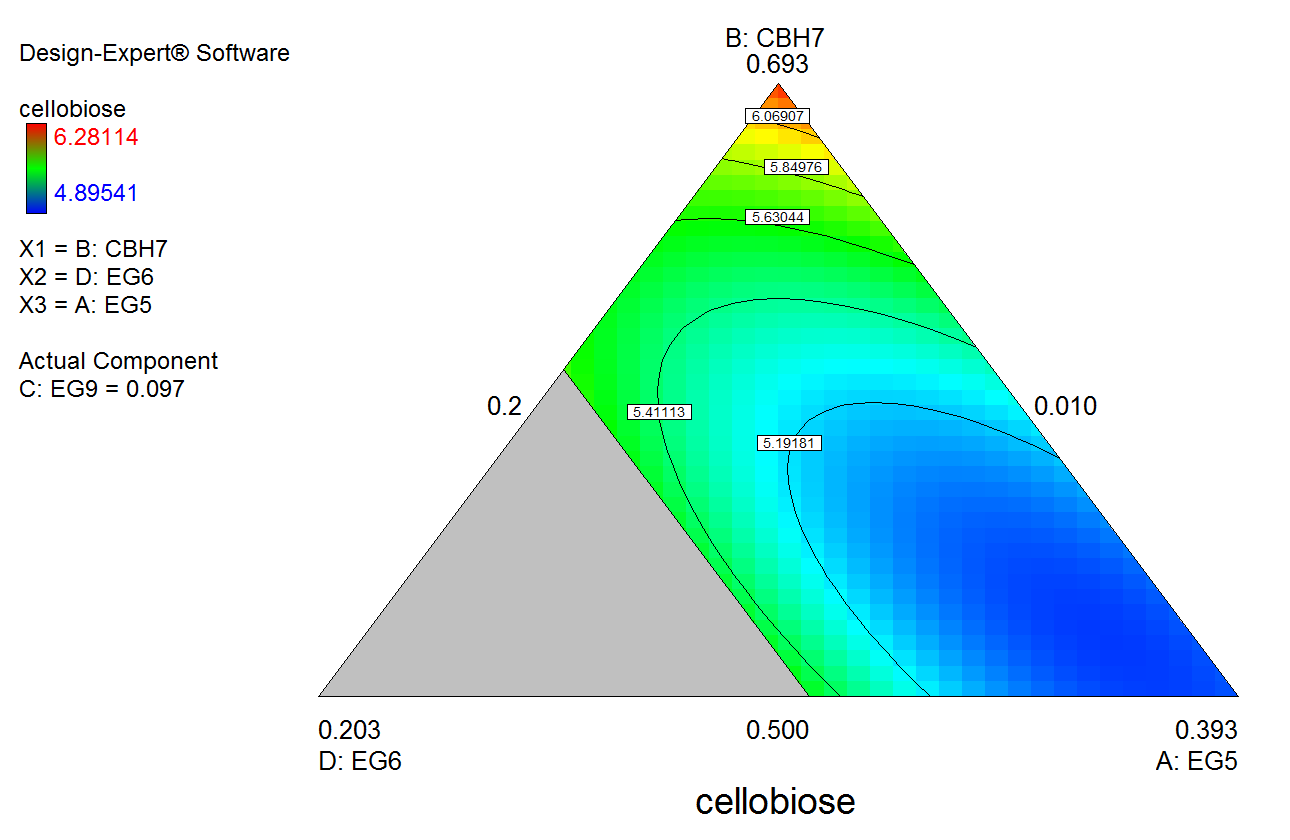
**

**(B) (i) (ii)**

**
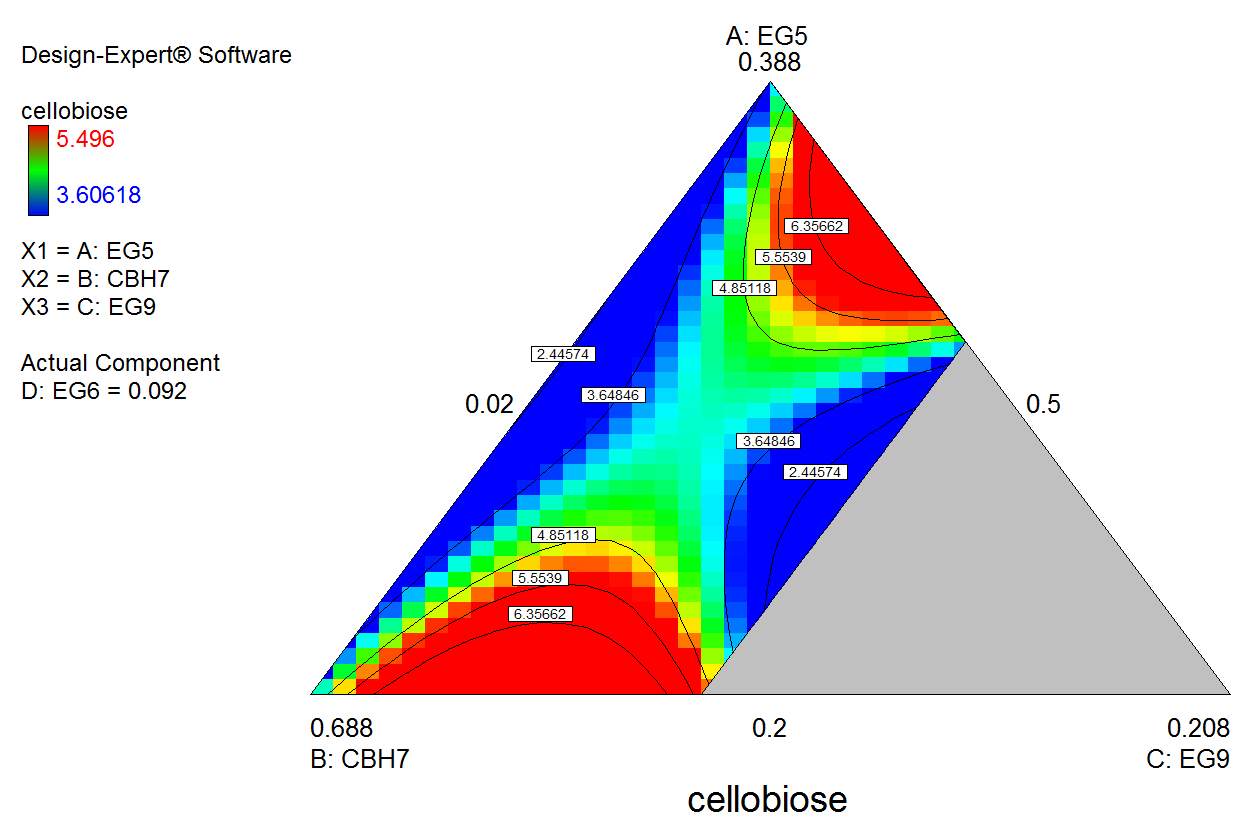

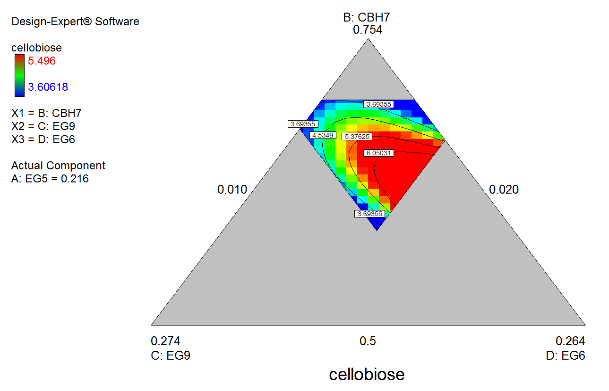
**

**(iii) (iv)**

**
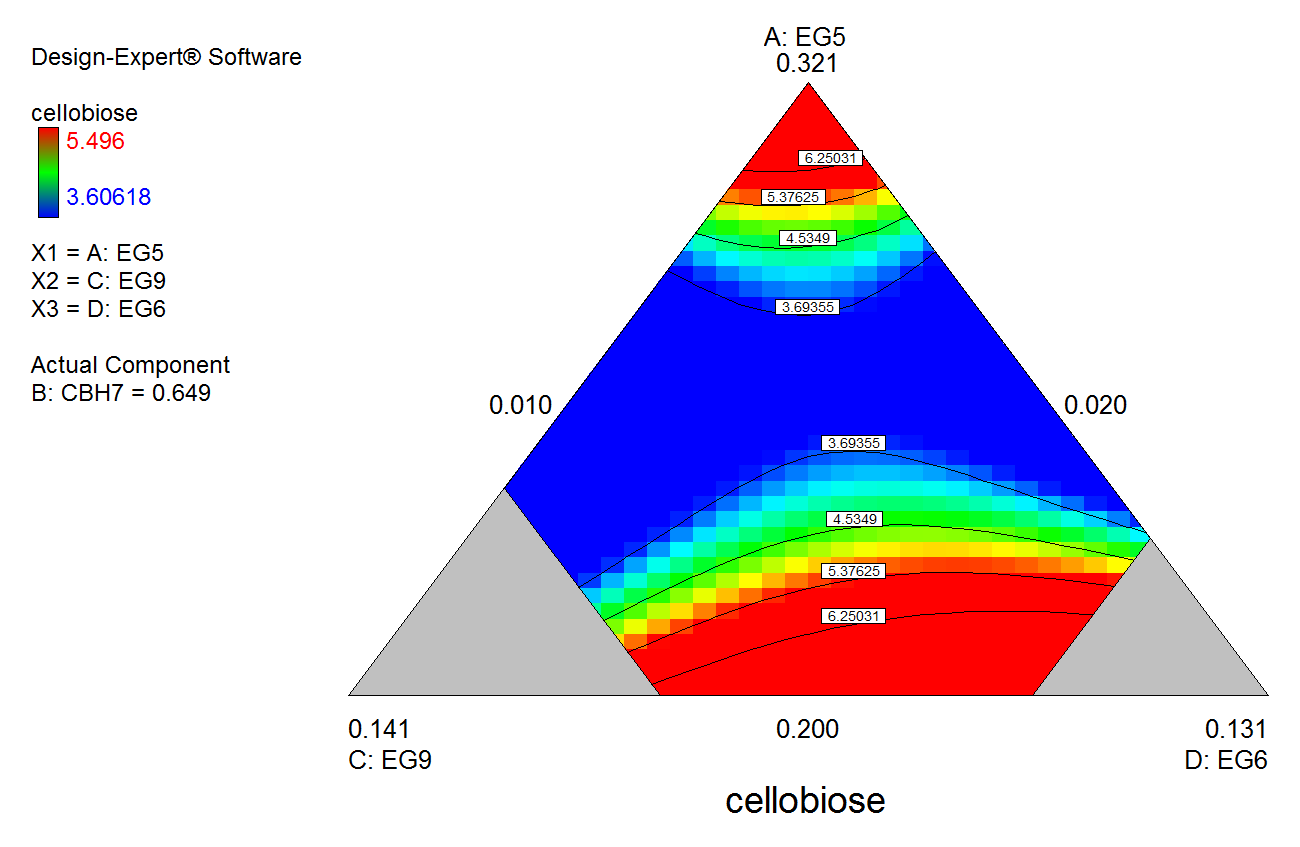

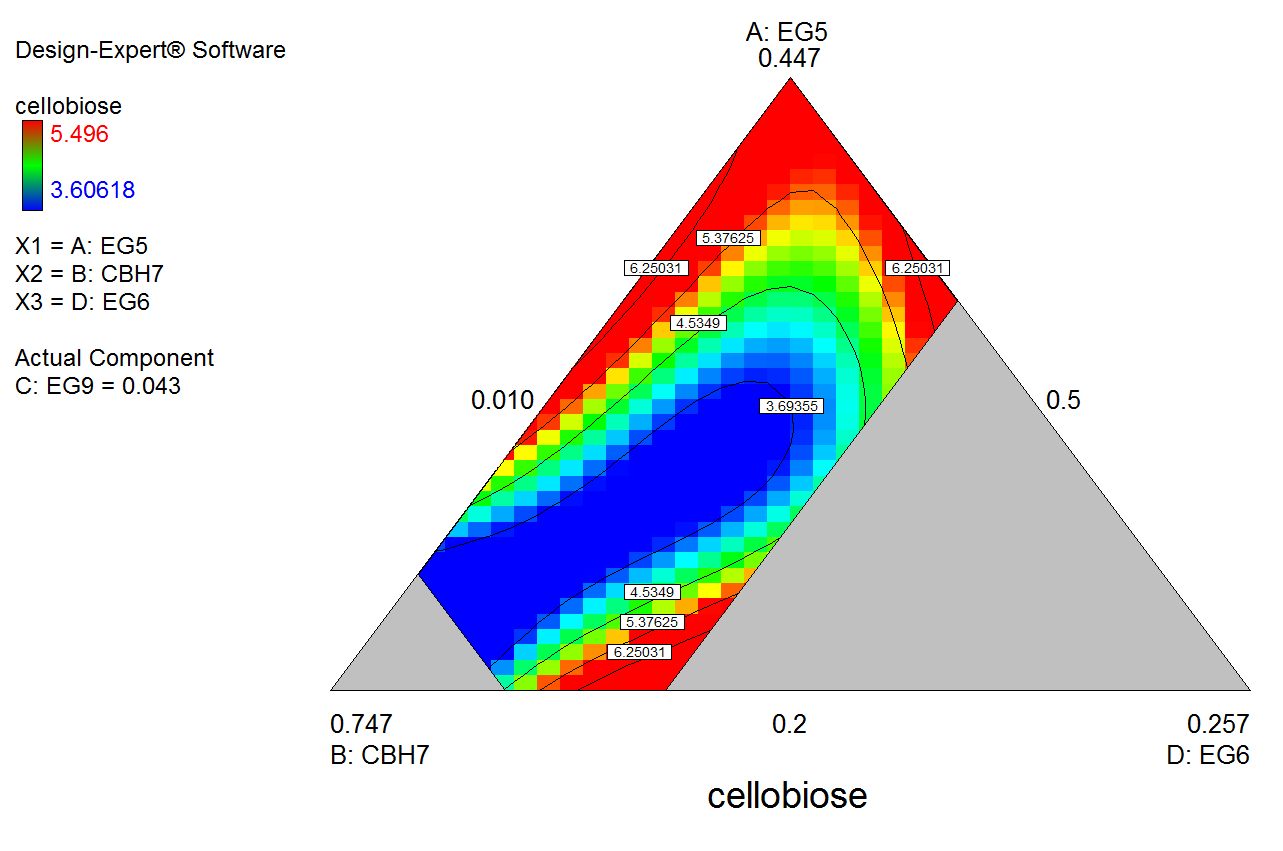
**
